# Supplementary material for: A surgically optimized intraoperative poly(I:C)-releasing hydrogel prevents cancer recurrence
Source: Cell Rep Med. 2023 Jul 18;4(7):101113. doi: 10.1016/j.xcrm.2023.101113 (PMC10394259; doi:10.1016/j.xcrm.2023.101113)
Supplement: Document S1. Figures S1–S6 [file mmc1.pdf]

Cell Reports Medicine, Volume *4*

Supplemental information

A surgically optimized intraoperative poly(I:C)-releasing hydrogel prevents cancer recurrence

Francois Xavier Rwandamuriye, Cameron W. Evans, Ben Wylie, Marck Norret, Breana Vitali, Diwei Ho, Dat Nguyen, Ellise A. Roper, Tao Wang, Matt S. Hepburn, Rowan W. Sanderson, Maren Pfirrmann, Vanessa S. Fear, Catherine A. Forbes, Ken Wyatt, Anne L. Ryan, Terrance G. Johns, Marianne B. Phillips, Rupert Hodder, Connull Leslie, Brendan F. Kennedy, Rachael M. Zemek, Killugudi Swaminathan Iyer, and Willem Joost Lesterhuis

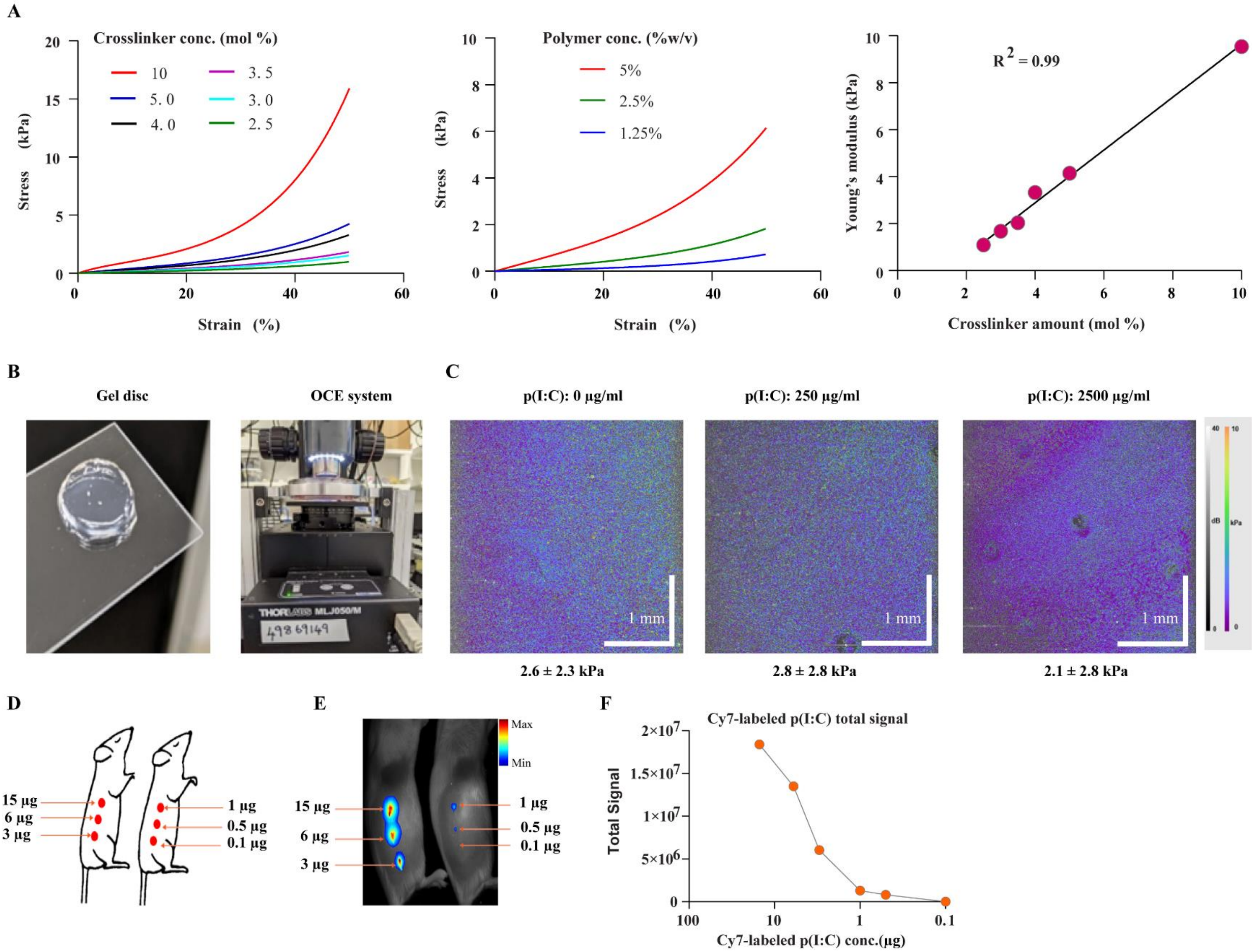

Figure S1. Characterization of HA hydrogels. Related to Figures 1 and 3.

(A) Stress-strain curves of different formulations of hydrogels with varying amount of DTPH crosslinker or hyaluronic acid polymer as well a the correlation of Young's modulus with an increased amount of cross-linker (mol %) while the amount of polymer is kept constant. The experiment was performed once. N = three replicates for each hydrogel.

(B) Sample of a gel disc and the optical coherence elastography system as previously described by Kennedy *et al*. [1].

(C) Representative quantitative micro-elastography (QME) scan of hydrogels with different amount of encapsulated poly(I:C), with stiffness calculated in kPa. Representative QME images of gels with poly(I:C) concentrations of 0 µg/ml, 250 µg/ml, and 2500 µg/ml, are shown in the lateral (xy) plane, acquired over a lateral field of view of 3 mm × 3 mm, approximately 200 µm below the top surface of the gel. The lateral voxel size of 3 µm × 3 µm resulted in 1,000,000 elasticity measurements in each 2-D image. In addition, the mean and standard deviation of all the elasticity measurements over the QME field of view was quantified for each poly(I:C) concentration. The elasticity mean ± standard deviation for the 0 µg/ml, 250 µg/ml, and 2500 µg/ml was 2.6 ± 2.3 kPa, 2.8 ± 2.8 kPa, and 2.1 ± 2.8 kPa, respectively. Data are presented as mean ± SD.

(D-F) *In vivo* quantification of Cy7 labeled poly(I:C). (D) Experimental design. Mice were injected s.c. with different amounts (final volume 50 µl) of Cy7-labeled poly(I:C), at adjacent points.

(E) Fluorescence IVIS imaging showing the Cy7 signal from labeled poly(I:C) injected in mice.

(F) Quantification of Cy7 signal showing the detection limit of Cy7-labeled poly(I:C).

The experiment was performed once. In (F), the Cy7 signal was quantified as total signal (x10^6 phot/cm2/s).

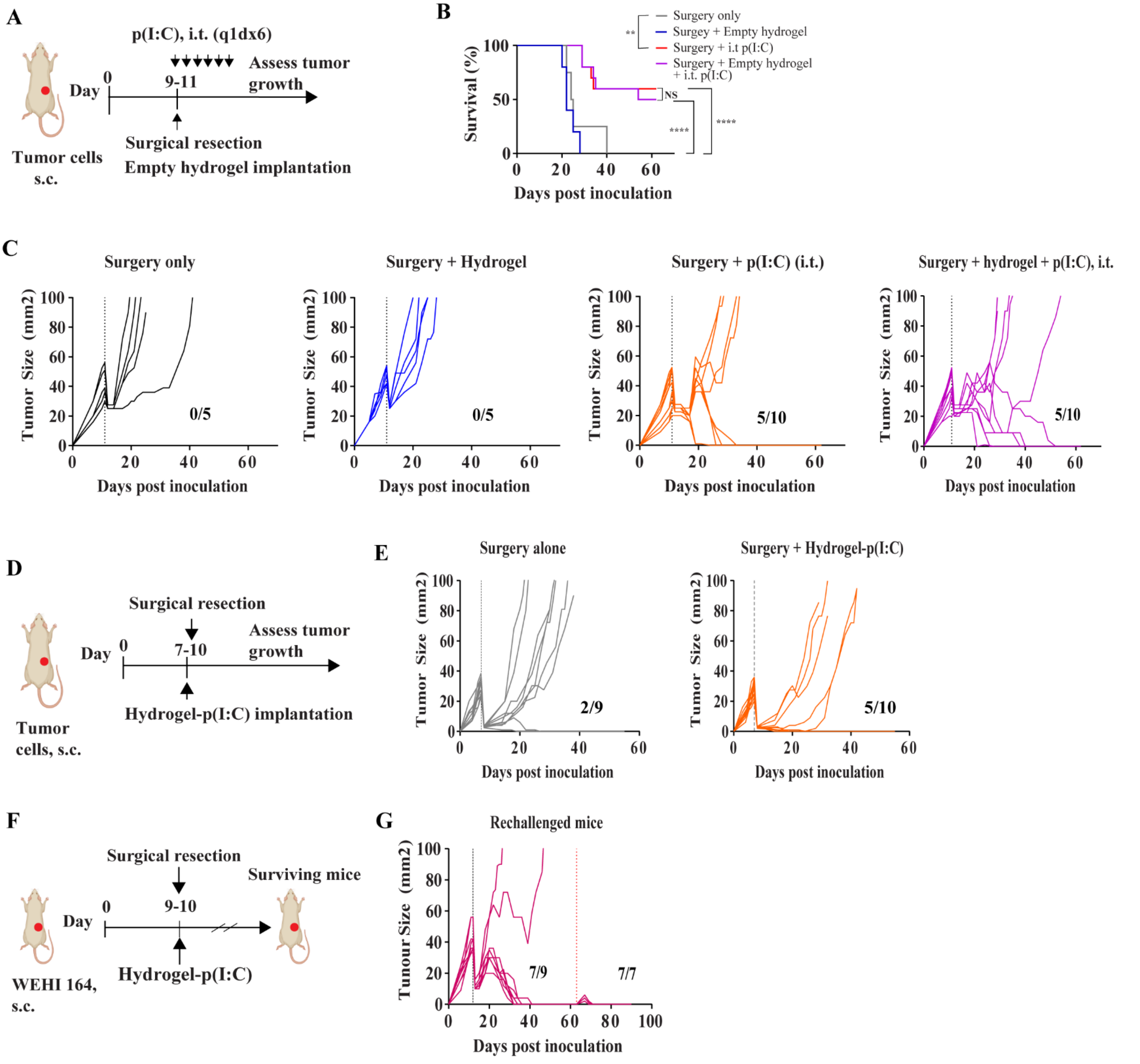

Figure S2. Efficacy of poly(I:C)-releasing hydrogel. Related to Figure 3.
(A and B) Intraoperative hydrogel application does not impair poly(I:C) efficacy**.** (A) Experimental design. Mice were inoculated s.c. with WEHI 164 tumor cells on the right flank. Once tumors were established, a 50% debulk of the tumor was performed and 100 µl of empty hydrogel was left in the resection site. Mice were dosed with poly(I:C) or vehicle, daily for 6 days (q1dx6) in the tumor/wound area.
(B and C) Survival curves (B) and tumor growth curves (C) of WEHI 164-bearing mice showing different treatment groups. The dashed line indicates the day of surgery. NS: non-significant. N = 5-10 mice per group. Statistical analyzes were performed using log-rank (Mantel-Cox) test to compare survival. The significance is represented with asterisks (*) according to the following values. *$P\leq 0.05$, **$P\leq 0.005$, ***$P\leq 0.0005$, ****$P\leq 0.0001$.
(D and E) Efficacy of intraoperative poly(I:C)-releasing hydrogel in M3-9-M tumor model**.** (D) Experimental design. Mice were inoculated with M3-9-M tumor cell line, s.c, on the right flank. Once tumors were established, a 90% debulk of tumor was performed and 100 µl of empty hydrogel or hydrogel-p(I:C) (250 µg) was left in the tumor resection site. (E) Tumor growth curves. The dashed line indicates the day of surgery. Data shown are from 2 independent experiments. N = 9-10 mice per group.
(F and G) The intraoperative poly(I:C)-releasing hydrogel induces a systemic immune memory response.
(F) Experimental design as in Figure 3D. Surviving, tumor-free, mice were rechallenged with WEHI 164 tumor cell line, s.c. on the left flank (four weeks after complete tumor regression).
(G) Tumor growth curves of surviving mice before and after rechallenge. The dashed black line indicates the day of surgery. The dashed red line indicates the day of rechallenge. Data shown is from one experiment, n = 7-9 mice per group.

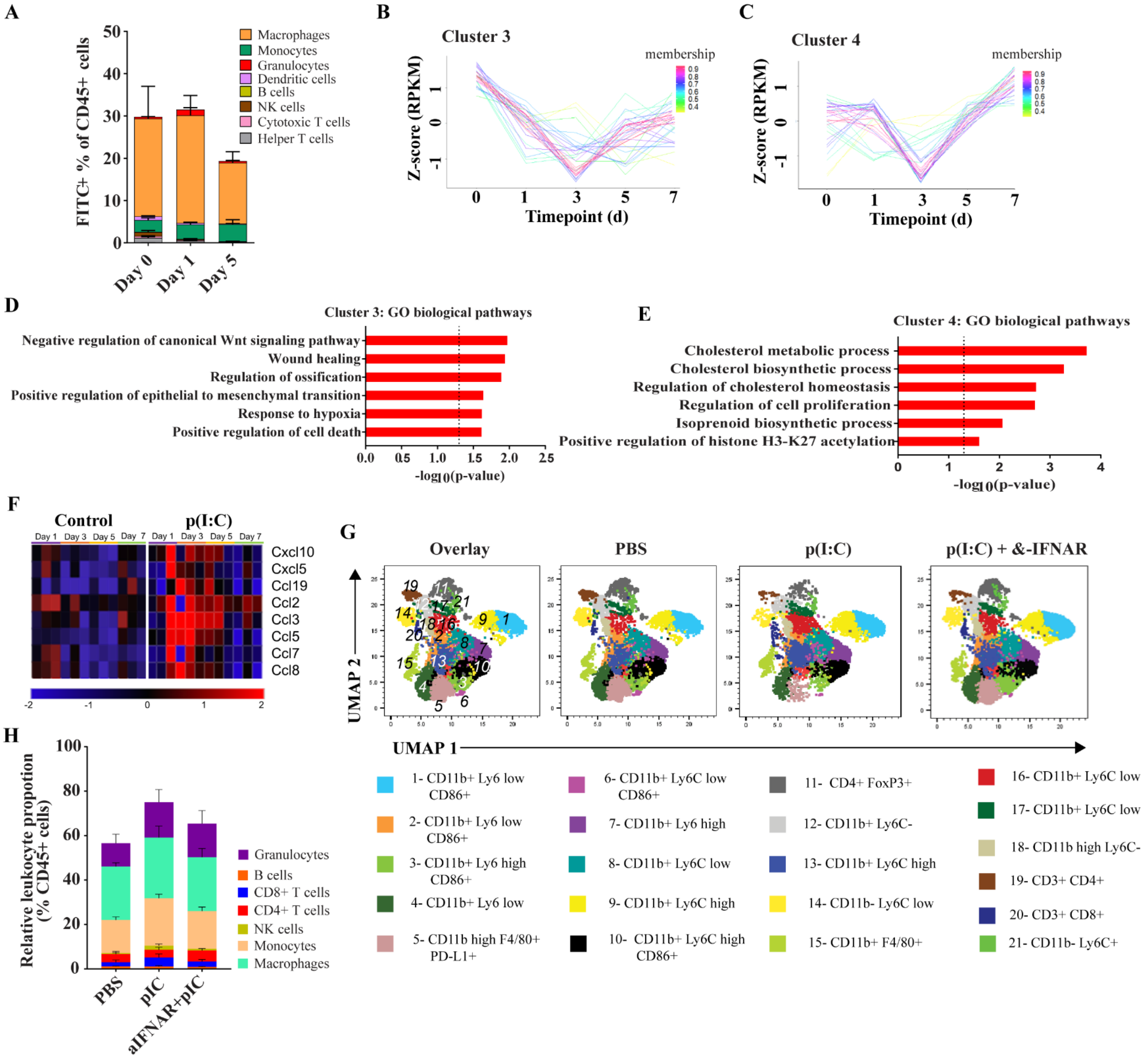

Figure S3. Prolonged poly(I:C) administration induces a transient IFNα response and reshapes the TME. Related to Figure 4.
(A) Uptake of poly(I:C) by immune cells. Experimental setup as in Figure 4A. Mice bearing WEHI 164 tumors were treated with poly(I:C), i.t., daily, for 4 days and a single injection of fluorescein-labeled poly(I:C) (50 ug), i.t., one hour before harvesting tumors for flow cytometry. Bar chart for FITC-labeled poly(I:C) detection in respective populations. N = 3 mice per group.
(B-E) Time-dependent analysis of gene expression in poly(I:C) or vehicle treated tumors. Experimental setup as in Figure 4C. Mice with established WEHI 164 tumors were treated with daily poly(I:C), 10 ug/day, or vehicle, i.t., for 6 days (q1dx6). Tumors were harvested on days 1, 3, 5, and 7, immediately placed in RNA later before subsequent RNA extraction for bulk RNA sequencing. N = 3 mice per group. (B) Time course variation in expression of gene clusters for Cluster 3. (C) Time course variation in expression of gene clusters for Cluster 4. TCseq analysis was used to cluster genes with similar expression over time.
(D) Top 5 upregulated biological pathways in Cluster 3.
(E) Top 5 upregulated biological pathways in Cluster 4.
(F) Poly(I:C) induces the expression of monocyte-attracting chemokines in the tumor microenvironment. Experimental setup as in Figure 4C. Heatmap of gene expression levels of monocyte-attracting chemokines in poly(I:C) and vehicle treated groups across the different time points. N = 3 mice per group.
(G) UMAP showing clustering of cell populations across different treatment groups.
(H) Relative proportion of different immune cells across different treatement groups.

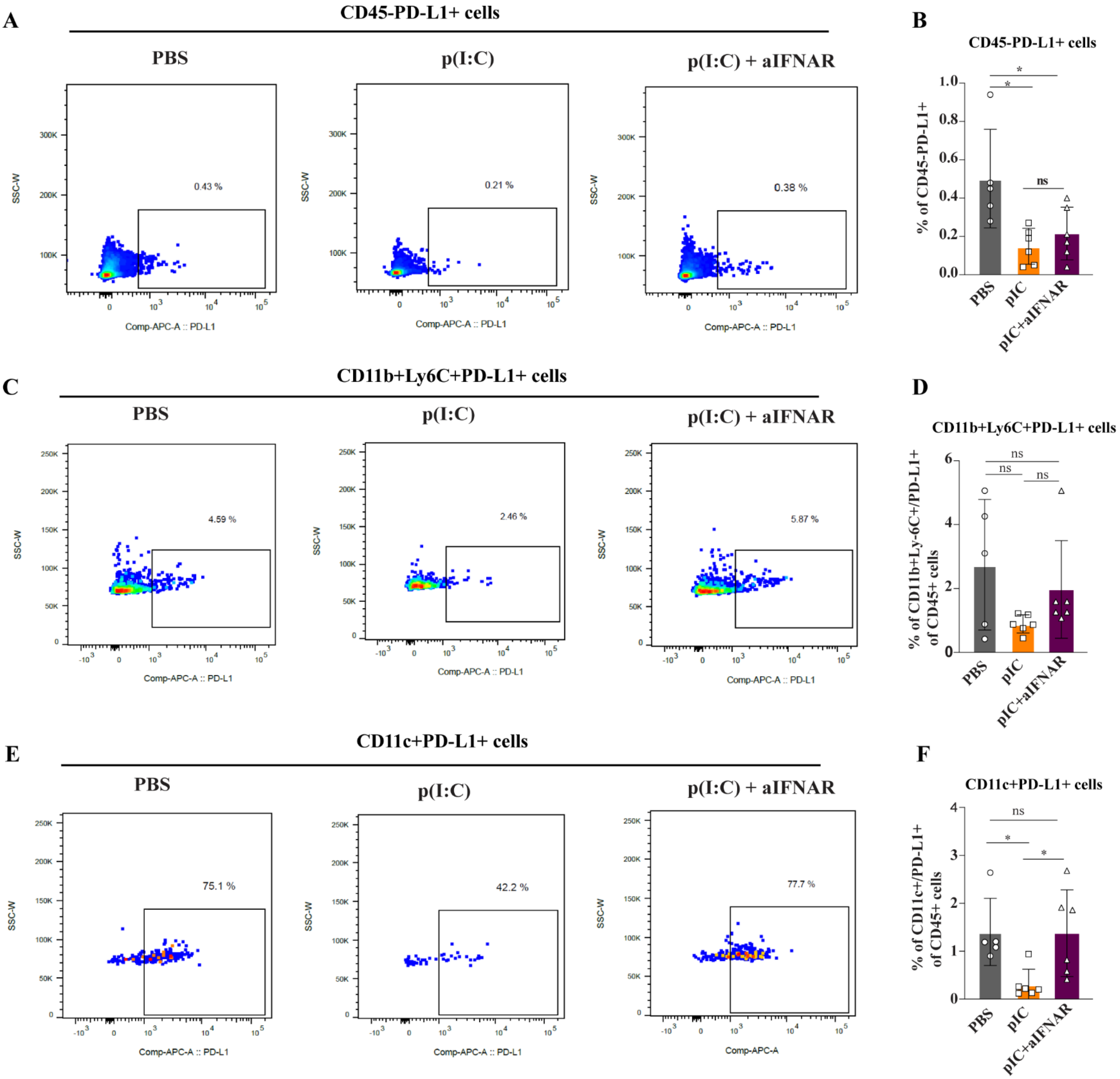

Figure S4. Expression of PD-L1 across different cell populations in the TME. Related to Figure 4 and Figure 5.
Experimental setup as in Figure 4I.
(A) Representative FACS plots of PD-L1 expression, gated on $CD45^-$/Live cells.
(B) Proportion (%) of $PD\text{-}L1^+$ cells within the $CD45^-$ cell fraction.
(C) Representative FACS plots of PD-L1 expression, gated on $CD11b^+Ly6C^+$ cells.
(D) Proportion (%) of $CD11b^+Ly6C^+$ $PD\text{-}L1^+$ cells within $CD45^+$ cell fraction.
(E) Representative FACS plots of PD-L1 expression, gated on $MHCII^+CD11c^+$ cells.
(F) Proportion (%) of $CD11c^+$ $PD\text{-}L1^+$ cells within $CD45^+$ cell fraction.
N = 5-6 biologically independent samples per group. Statistical analyzes were performed with one-way ANOVA followed by Tukey's multiple comparison test, ns = not significant. The significance is represented with asterisks (*) according to the following values: *P≤ 0.05, **P≤ 0.005, ***P≤ 0.0005, ****P≤ 00001.

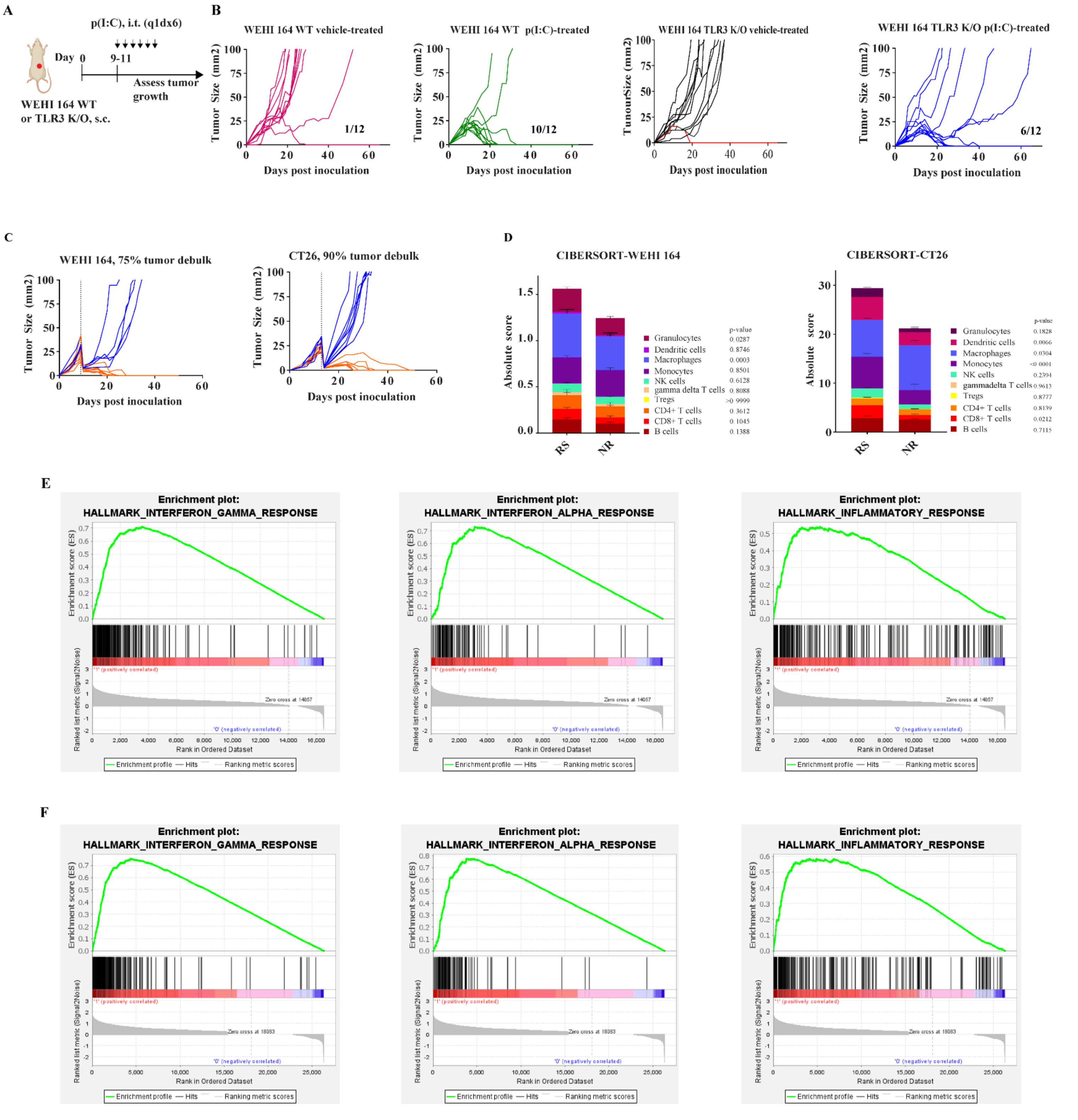

Figure S5. A pre-existing IFN gene signature predicts response to poly(I:C) hydrogel. Related to Figure 5.
(A and B) Efficacy of poly(I:C) in tumors derived from TLR3 K/O WEHI 164 cells. (A) Experimental setup**.** Mice were inoculated with either wild type WEHI 164 or TLR3 K/O WEHI 164 cells, s.c, on the right flank. Mice with established tumors were treated with poly(I:C), 10 µg/day for 6 days (q1dx6). (B) Tumor growth curves of TLR3 K/O WEHI 164- or WEHI 164 WT-bearing mice. Data shown are pooled from 2 independent experiments, n = 8-10 mice per group. WT: wild type. TLR3 K/O: Toll like receptor 3 knock out.
(C) Tumor growth curves and CIBERSORT plots of responders and non-responders in WEHI 164 and CT26 tumors treated with poly(I:C) hydrogel, allowing classification into responders (orange) or non-responders (blue). N = 9-10 mice per treated group.
(D) CIBERSORT plots from RNAseq data of responsive and non-responsive WEHI 164 and CT26 tumors. N = 4-6 biologically independent samples per group. For CIBERSORT data, statistical analysis was performed using two-way ANOVA with Benjamini and Hochberg correction for multiple comparisons.
(E and F) Responsive tumors are characterized by an IFN-active, inflammatory microenvironment. Experiment setup as in Figure 5C. (E) GSEA plots comparing responsive versus non-responsive WEHI 164 tumors. N = 5 biologically independent samples per group. (F) GSEA plots comparing responsive versus non-responsive CT26 tumors. N = 4-6 biologically independent samples per group. The experiment was performed once. GSEA software was used to analyze normalized gene expression data to compare responders and non-responders.

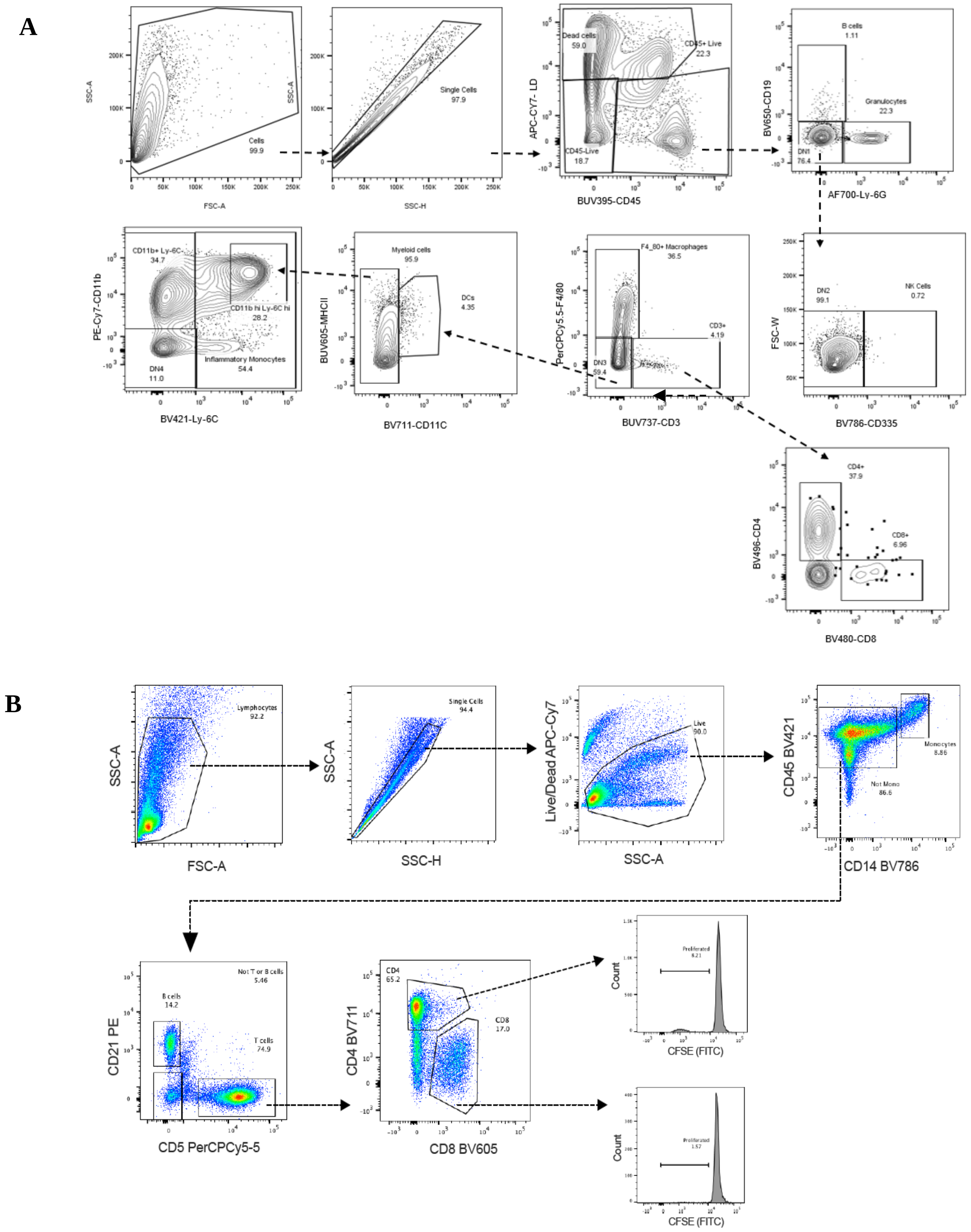

Figure S6. Gating strategies. Related to Figure 4 and Figure 6.
(A) Gating strategy for murine immune cell populations. Live $CD45^+$ cells, DN1 (excludes $CD19^+$ B cells, $Ly6G^+$ Granulocytes), DN2 (excludes $CD335^+$ NK cells), F4/80+ macrophages, $CD11c^+$ DCs, $CD11b^+$ $Ly6^{hi}$ cells, $CD3^+$ cells, $CD4^+$ and $CD8^+$ T cells gating strategies are indicated.
(B) Gating strategy for canine immune cell populations. Live $CD45^+$ cells, $CD4^+$ and $CD8^+$ T cells, and CFSE proliferation gating strategies are indicated.

Supplemental Reference

1. Kennedy, K.M., Chin, L., McLaughlin, R.A., Latham, B., Saunders, C.M., Sampson, D.D., and Kennedy, B.F. (2015). Quantitative microelastography: Imaging of tissue elasticity using compression optical coherence elastography. Sci. Rep. 5, 15538. https://doi.org/10.1038/srep15538.
