# Supplementary material for: A surgically optimized intraoperative poly(I:C)-releasing hydrogel prevents cancer recurrence
Source: Cell Rep Med. 2023 Jul 18;4(7):101113. doi: 10.1016/j.xcrm.2023.101113 (PMC10394259; doi:10.1016/j.xcrm.2023.101113)
Supplement: Document S2. Article plus supplemental information [file mmc8.pdf]

Cell Reports
Medicine

Article

A surgically optimized intraoperative poly(I:C)-releasing hydrogel prevents cancer recurrence

Graphical abstract

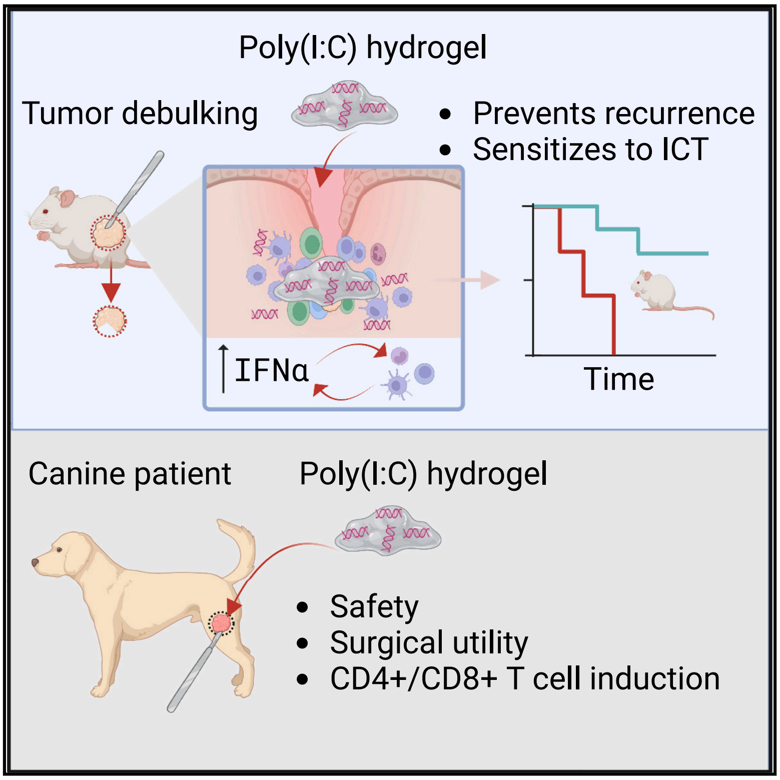

Highlights

- A surgically optimized poly(I:C) hydrogel prevents postoperative cancer recurrence
- Response is IFNα dependent, attracting inflammatory monocytes and depleting Tregs
- An IFN signature predicts the response, and the hydrogel sensitizes tumors to ICT
- Safety, surgical utility, and T cell activation are established in pet dogs with cancer

Authors

Francois Xavier Rwandamuriye, Cameron W. Evans, Ben Wylie, ..., Rachael M. Zemek, Killugudi Swaminathan Iyer, Willem Joost Lesterhuis

Correspondence

swaminatha.iyer@uwa.edu.au (K.S.I.), willem.lesterhuis@uwa.edu.au (W.J.L.)

In brief

Rwandamuriye et al. develop a hydrogel to slowly release poly(I:C) in the tumor resection area. The hydrogel prevents tumor recurrence in multiple mouse models and sensitizes tumors to immune checkpoint therapy. Its safety, surgical utility, and immunogenicity are confirmed in a veterinary trial in canine patients with soft tissue tumors.

Rwandamuriye et al., 2023, Cell Reports Medicine *4*, 101113
July 18, 2023 © 2023 The Author(s).
https://doi.org/10.1016/j.xcrm.2023.101113

CellPress

Cell Reports Medicine

CellPress
OPEN ACCESS

Article

A surgically optimized intraoperative poly(I:C)-releasing hydrogel prevents cancer recurrence

Francois Xavier Rwandamuriye,[1] Cameron W. Evans,[2,13] Ben Wylie,[1,13] Marck Norret,[2] Breana Vitali,[1] Diwei Ho,[2] Dat Nguyen,[1] Ellise A. Roper,[1] Tao Wang,[1] Matt S. Hepburn,[3,4,5] Rowan W. Sanderson,[3,4,5] Maren Pfirrmann,[1,6] Vanessa S. Fear,[1] Catherine A. Forbes,[1] Ken Wyatt,[7,8] Anne L. Ryan,[1,9] Terrance G. Johns,[1] Marianne B. Phillips,[1,9] Rupert Hodder,[10] Connull Leslie,[11] Brendan F. Kennedy,[3,4,5] Rachael M. Zemek,[1] Killugudi Swaminathan Iyer,[2,12,*] and Willem Joost Lesterhuis[1,12,14,*]

[1]Telethon Kids Institute, The University of Western Australia, Nedlands, WA, Australia
[2]School of Molecular Sciences, The University of Western Australia, Crawley, WA, Australia
[3]BRITElab, Harry Perkins Institute of Medical Research, QEII Medical Centre, Nedlands, WA, Australia
[4]Centre for Medical Research, The University of Western Australia, Crawley, WA, Australia
[5]Department of Electrical, Electronic and Computer Engineering, School of Engineering, The University of Western Australia, Crawley, WA, Australia
[6]Department of Medical BioSciences, Radboud University Medical Centre, Nijmegen, the Netherlands
[7]Perth Veterinary Specialists, Osborne Park, WA, Australia
[8]Murdoch Veterinary School, Murdoch University, Murdoch, WA, Australia
[9]Department of Oncology, Hematology and Tissue and Cellular Therapies, Perth Children's Hospital, Perth, WA, Australia
[10]Department of Surgery, Sir Charles Gairdner Hospital, Nedlands, WA, Australia
[11]Department of Anatomical Pathology, PathWest Laboratory Medicine, QEII Medical Centre, Nedlands, WA, Australia
[12]Senior author
[13]These authors contributed equally
[14]Lead contact
*Correspondence: swaminatha.iyer@uwa.edu.au (K.S.I.), willem.lesterhuis@uwa.edu.au (W.J.L.)
https://doi.org/10.1016/j.xcrm.2023.101113

SUMMARY

Recurrences frequently occur following surgical removal of primary tumors. In many cancers, adjuvant therapies have limited efficacy. Surgery provides access to the tumor microenvironment, creating an opportunity for local therapy, in particular immunotherapy, which can induce local and systemic anti-cancer effects. Here, we develop a surgically optimized biodegradable hyaluronic acid-based hydrogel for sustained intraoperative delivery of Toll-like receptor 3 agonist poly(I:C) and demonstrate that it significantly reduces tumor recurrence after surgery in multiple mouse models. Mechanistically, poly(I:C) induces a transient interferon alpha (IFNα) response, reshaping the tumor/wound microenvironment by attracting inflammatory monocytes and depleting regulatory T cells. We demonstrate that a pre-existing IFN signature predicts response to the poly(I:C) hydrogel, which sensitizes tumors to immune checkpoint therapy. The safety, immunogenicity, and surgical feasibility are confirmed in a veterinary trial in canine soft tissue tumors. The surgically optimized poly(I:C)-loaded hydrogel provides a safe and effective approach to prevent cancer recurrence.

INTRODUCTION

Surgery remains the cornerstone of treatment for many solid tumors, frequently in combination with (neo-)adjuvant chemotherapy and/or radiotherapy.[1] Despite these treatments, recurrence remains a major cause of death in many cancer types.[2] Adjuvant or neo-adjuvant immunotherapy with immune checkpoint antibodies has improved recurrence-free survival in some cancers such as melanoma[3] and non-small cell lung cancer[4] but has been less successful in other cancers.[5] In addition, the required systemic administration exposes all organ systems to these antibodies, which results in frequent and significant toxicity.[6] There is a strong clinical need for more effective and potentially safer treatment options to prevent post-surgical cancer recurrence.

Surgery to resect the primary tumor provides an opportunity to access and target the tumor microenvironment (TME). Recent studies have shown that biomaterials can be applied during surgery to deliver therapies.[7,8] Hydrogels, in particular, offer an opportunity for safe, targeted, and sustained delivery of immunotherapy as they have an excellent safety profile,[9,10] and can be easily combined with systemic therapies.[11] However, several important aspects of intraoperative immunotherapy-releasing hydrogels have so far not been adequately addressed. These include the optimal physical properties of the hydrogel, allowing the hydrogel to be applied at areas of positive tumor margins; the

This is an open access article under the CC BY license (http://creativecommons.org/licenses/by/4.0/).

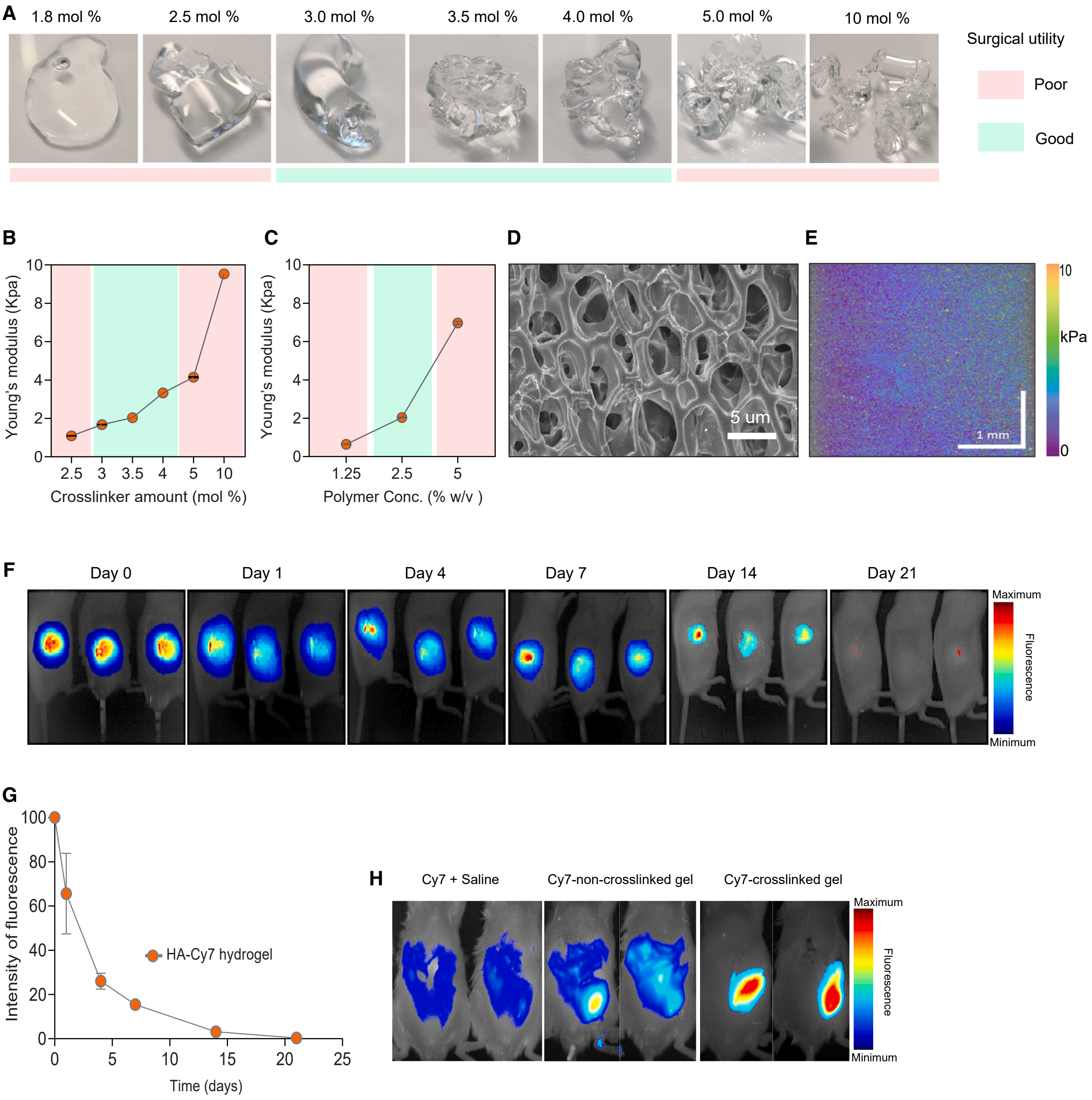

Figure 1. Design and characterization of a surgically optimized hyaluronic acid-based hydrogel

(A) Representative photographs of different hydrogel formulations obtained using a constant percentage of HA polymer (2.5% w/v) and varying amounts of crosslinker (1.8–10 mol %).

(B and C) Young's modulus for different hydrogel formulations with varying amounts of DTPH crosslinker (B) or HA polymer (C). Data are mean ± SEM; n = 3 replicates for each hydrogel. Experiments were performed more than twice in (A) and once in (B) and (C). See also Figure S1A.

(D) Scanning electron microscopy (SEM) image of the optimized hydrogel (2.5% w/v HA polymer and 3.5 mol % crosslinker). Scale bar, 5 μM.

(E) Quantitative micro-elastography (QME) scan of the optimized hydrogel. Scale bar, 1 mm. Volumetric QME scans were acquired over a 3 × 3 × 2.5 mm (*x*, *y*, *z*) field of view in the center of HA gel disc with a voxel size of 3 × 3 × 2.5 μm (*x*, *y*, *z*). This voxel size resulted in 1 million elasticity measurements in each two-dimensional (2D) image. Experiments were performed once in (E). See also Figure S1B.

(F) Representative *in vivo* fluorescence images for gel degradation. HA hydrogels were labeled with Cy7; n= 3 mice per group.

(legend continued on next page)

optimal duration and dosing of the immunotherapy, knowing that immunotherapy often does not display a linear dose-response correlation[12]; the potential interference with post-surgical wound healing by either the hydrogel or the immunotherapeutic drug; and combination with standard systemic immunotherapy. Here, we set out to develop a safe and effective, surgically optimized hydrogel for sustained release of immunotherapy following intraoperative application during oncological surgery.

RESULTS

Design and characterization of a surgically optimized hyaluronic acid-based hydrogel

We chose hyaluronic acid (HA)-based hydrogels because of their excellent biocompatibility and biodegradability.[13] HA has been extensively used in a variety of medical applications in tissue engineering[14] and cosmetic surgery.[15] Importantly, HA has proven beneficial effects on wound healing,[16] leading to its use in the treatment of chronic venous wounds[17] and oral ulcers.[18] HA-based hydrogels were prepared using 3,3′-dithiobispropanoic dihydrazide (DTPH) crosslinker.[19]

To identify a hydrogel with the desired physical and mechanical characteristics for intraoperative application, we tested the effect of HA concentration and crosslinker amount on the physical properties of the hydrogel. We assessed the surgical utility by visual inspection following expulsion from a syringe (Video S1), and by manually manipulating the hydrogels to assess stiffness (Figure 1A). In addition, we tested selected hydrogels *in vivo* for surgical utility, by applying them in a subcutaneous surgical site in mice. We found that hydrogels with 2.5% w/v HA concentration and 3–4 mol % crosslinker had an optimal consistency (Video S1) that allowed for easy and even application in the surgical resection cavity (Video S2), including in small anatomical pockets. Hydrogels of this composition adhered well to the different tissues and did not leak out after wound closure (Video S2). In contrast, hydrogels with 1.25% w/v HA and 3.5 mol % crosslinker were not solid, resulting in the gel leaking from the wound (Video S3), whereas the hydrogels with 5% w/v HA polymer and 3.5 mol % crosslinker were too stiff and brittle (Video S4), which made them difficult to apply evenly, particularly in small anatomical locations, and they did not adhere well to the tissue (Video S5).

Having identified the optimal gel composition for surgical use, we quantified the mechanical properties of the hydrogels using uniaxial compression testing, which showed that increasing either the amount of crosslinker or the polymer concentration resulted in increased stiffness and Young's modulus (Figure S1A), as quantified by Young's modulus (Figures 1B and 1C). The optimal hydrogels had a Young's modulus of 1.7–3.3 kPa (Figure 1B).

An intraoperatively applied hydrogel for drug delivery requires a homogeneous structure and consistent physical characteristics throughout the gel to ensure reliable and reproducible drug release across the wound bed. The mesh-like porous structure of the hydrogel was confirmed by scanning electron microscopy (Figure 1D). In addition, we assessed the mechanical properties of hydrogels on the microscale using quantitative micro-elastography (QME)[20] (Figures S1B), which showed a uniform microscale stiffness across the gel surface (Figure 1E).

Chemically crosslinked, drug-loaded hydrogels can release their therapeutic cargo through different mechanisms including hydrogel swelling, drug diffusion, surface and bulk erosion,[10] enzymatic digestion, and chemical degradation.[21] As the exact *in vivo* levels of hyaluronidases in the context of a healing wound are unknown, it is impossible to faithfully represent the degradation kinetics *in vitro*. We therefore assessed the *in vivo* degradation of the hydrogel by implanting a Cy7-labeled hydrogel in a subcutaneous wound in mice and measured the degradation rate of the gel *in vivo* using fluorescence imaging. We found that the optimized hydrogel provided consistent and prolonged degradation over more than two weeks following application in the wound area (Figures 1F and 1G).

A last important requirement for a drug-releasing gel applied in a surgical wound cavity is for it to remain *in situ* when the patient regains mobility after surgery, particularly when applied at potentially compromised tumor margins. We therefore assessed the mobility of the gel in mice by implanting a Cy7-labeled hydrogel intraperitoneally to allow free movement. We found that an uncrosslinked Cy7-labeled gel or Cy7 dissolved in saline disseminated rapidly over the peritoneal cavity, while the crosslinked hydrogel stayed close to the implantation site, confirming its anatomical anchoring following application (Figure 1H). Together, these data show that the surgically optimized hydrogel with 2.5% w/v HA and 3.5 mol % DTPH crosslinker is slowly degraded in a healing wound, while remaining at its anatomical location following application, and thus forms an ideal biomaterial for intraoperative immunotherapy release.

Prolonged intratumoral poly(I:C) treatment is effective and safe at low dose

Next, we aimed to determine the optimal cancer immunotherapy to incorporate into the gel for intraoperative application, considering both anti-cancer efficacy as well as potential wound healing interference. Given recent evidence that type I interferon (IFN) is crucial for wound healing[22] as well as anti-cancer immunity,[23,24] we focused on immunotherapeutics that induce type I IFNs. First, we screened recombinant IFNβ, recombinant IFNα, the Toll-like receptor (TLR) 3 agonist polyinosinic:polycytidylic acid (poly[I:C]), and the stimulator of IFN genes (STING) agonist, 5,6-dimethylxantenone-4-acetic acid (DMXAA) for their ability to induce local tumor control. As intraoperative immunotherapy has the advantage that it is applied at the effector site without needing to achieve high systemic and potentially toxic exposure levels, we sought to use relatively low doses of these drugs. As a

(G) *In vivo* gel degradation profile. Cy7 signal as total signal ($\times 10^6$ phot/cm^2/s). Data are mean ± SD; n = 3 mice per group. Experiments were performed twice in experiments (F) and (G).

(H) *In vivo* mobility of hydrogels. *In vivo* fluorescence images of free Cy7 resuspended in saline, a non-crosslinked Cy7-labeled HA hydrogel (2.5% w/v HA, non-crosslinked) or the crosslinked hydrogel (2.5% w/v HA, 3.5 mol % DTPH) labeled with Cy7, imaged at 3 h after intraperitoneal injection in mice; n = 2 mice per group. The experiment was performed once.

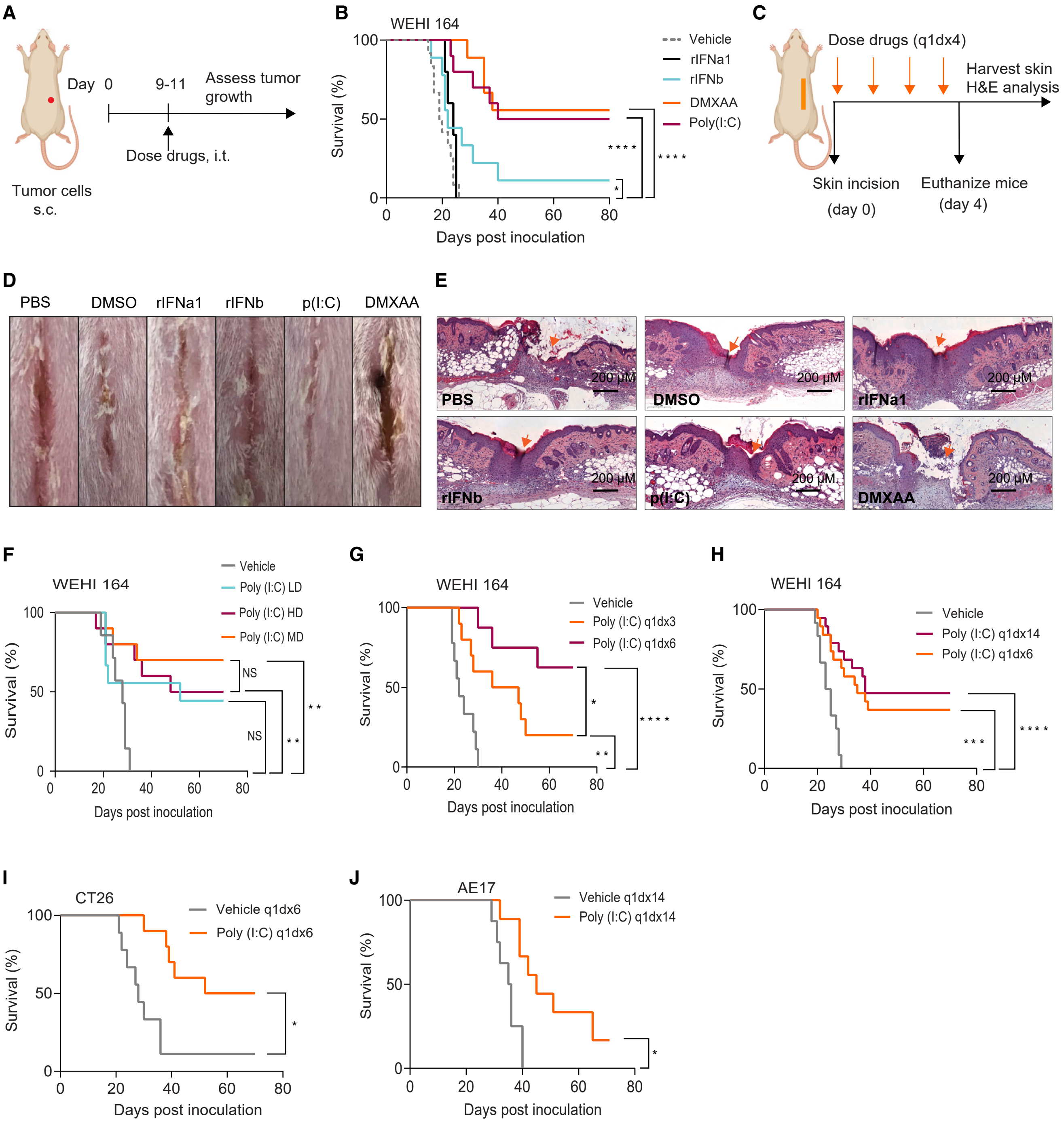

Figure 2. Prolonged intratumoral poly(I:C) treatment is effective and safe at low dose

(A) Experimental design. Mice with established tumors were treated intratumorally (i.t.) with immune adjuvants, daily, for 3, 6, or 14 days, depending on the treatment schedule.

(B) Survival curves of WEHI 164-bearing mice treated with poly(I:C) 10 μg/day, DMXAA 50 μg/day, rIFNα 2,000 IU/day, rIFNβ 2,000 IU/day, or vehicle daily for 6 days; n = 8–10 mice per group.

(C) Experimental setup for the skin incision model. Mice were dosed with poly(I:C), DMXAA, rIFNα, or rIFNβ in the wound area, daily, for 4 days, using the same doses as in (B); n = 3–5 mice per group.

(D and E) Representative photographs of mice showing macroscopic wound healing (D) and H&E staining of skin cross-sections around the wounded area (E) collected on day 4 post-surgery. Scale bar, 200 μm. Magnification, 20×.

(legend continued on next page)

starting point, we chose 20% of the commonly used systemic doses in pre-clinical models on the basis of the literature.[25–29] We found that both poly(I:C) and DMXAA treatment resulted in significant regression of established tumors ($p < 0.0001$), while recombinant IFNβ had only a modest effect and recombinant IFNα1 had no effect (Figures 2A and 2B).

Next, we assessed the impact of these immune adjuvants on the wound healing response by using a full-thickness skin incision model (Figure 2C). Macroscopic observation of wounds treated with local STING agonist DMXAA showed unhealed wounds at day 4 post-surgery (Figure 2D). Microscopic analysis showed reduced wound closure with enhanced stromal ground substance formation compared with its vehicle control (Figure 2E). In contrast, local treatment with poly(I:C), as well as IFNα or IFNβ, did not affect wound healing negatively (Figures 2D and 2E).

We then compared the anti-tumor efficacy of varying doses of poly(I:C), by using 2% (0.05 mg/kg; low dose), 20% (0.5 mg/kg; medium dose), and 100% (2.5 mg/kg; high dose) of the reported systemic doses.[25–27] We delivered immunotherapy locally after partial tumor debulking surgery to mimic incomplete resection as occurs in patients.[30] Poly(I:C) was highly effective in preventing tumor relapse in WEHI 164 fibrosarcoma, with recurrence-free rates of 50%–70% ($p < 0.05$; Figure 2F). Although medium-dose levels appeared to provide the best outcome, there was no significant difference in survival across the three different doses (Figure 2F). These data confirm a relatively flat dose-response relationship for poly(I:C) and indicate that local administration of poly(I:C) can be given at a relatively low dose without losing efficacy in this surgical context.

Next, we determined the optimal duration of poly(I:C) treatment and found that daily injections for three days were less effective than daily injections for six days ($p < 0.05$; Figure 2G). Further increasing to fourteen daily injections showed no further benefit ($p = 0.49$; Figure 2H), demonstrating that the optimal treatment period for low dose local poly(I:C) is 1–2 weeks. We confirmed the efficacy of intratumoral (i.t.) poly(I:C) at this optimized dose and schedule in two additional tumor models, the moderately immunogenic colorectal cancer CT26 (Figure 2I) and the immunotherapy-resistant mesothelioma AE17[23,24] (Figure 2J). Taken together, these results demonstrate that poly(I:C) is highly efficacious when administered i.t. for a period of 1–2 weeks at low dose while also improving surgery-induced wound healing.

An intraoperative poly(I:C)-releasing hydrogel prevents tumor recurrence

We then proceeded to incorporate poly(I:C) in the gel. First, we queried whether the hydrogel could result in extended release of the drug. *In vitro* release profiling showed that the poly(I:C) release was dependent on hyaluronidase activity (Figure 3A). Second, we tested whether the addition of different concentrations of poly(I:C) had an effect on the mechanical properties of the HA gel, using QME.[20] We found that poly(I:C) was evenly distributed within the gel and did not change its stiffness (Figure S1C). Third, we assessed the *in vivo* release period of the poly(I:C) using Cy7-labeled poly(I:C) encapsulated in the hydrogel and applied in a subcutaneous surgical site in mice. We found that the signal was still detected at 3 weeks postinjection (Figures 3B, 3C, and S1D–S1F), with the majority (about 70%) of the drug being released in the first week (Figure 3C). Fourth, since hyaluronidase levels are increased during inflammation,[31] we queried whether the presence of poly(I:C)-induced inflammation influenced the degradation rate of the HA-based hydrogel. We used a Cy7-conjugated version of the HA hydrogel with unlabeled poly(I:C). Fluorescence imaging showed that the degradation rate was identical to a gel that did not contain poly(I:C) (Figures 3B and 3C). Together, these data demonstrate that the optimized HA hydrogel released the majority of the poly(I:C) over 1–2 weeks, which we earlier demonstrated was optimal for tumor control with low dose local poly(I:C) therapy.

As different studies have shown that HA oligomers can have tumor-promoting effects,[32] we tested whether the presence of the HA hydrogel could impair the efficacy of local poly(I:C) administration. We implanted an empty HA hydrogel in the resection cavity after partial tumor debulking and subsequently treated mice with i.t. poly(I:C) for six days by i.t. injection. We found that the HA hydrogel did not affect the efficacy of poly(I:C) (Figures S2A–S2C).

We next evaluated the efficacy of the poly(I:C)-releasing hydrogel in preventing local recurrence in the WEHI 164 model of incomplete tumor resection.[30] The extended release of poly(I:C) from the hydrogel, implanted in the tumor resection site, prevented local tumor recurrence in the majority of mice and resulted in a significant survival benefit compared with an empty hydrogel (60% vs. 0%; $p < 0.005$; Figure 3E). We confirmed these results in the less immunogenic models CT26 (Figure 3F) and M3-9-M (Figures S2D and S2E). We removed slightly more tumor bulk in these experiments (90% instead of 75%) as the CT26 colorectal cancer and M3-9-M rhabdomyosarcoma models grow more rapidly and are less immunogenic than the WEHI 164 fibrosarcoma model. Finally, we rechallenged surviving mice with the same tumor cell line on the opposite flank and found that all surviving mice were protected after distant rechallenge (Figure S2F and S2G). These results demonstrate that the local therapy with poly(I:C) hydrogel had induced systemic control and immune memory. Together, these data show that the optimized intraoperative poly(I:C)-releasing hydrogel prevents

(F) Survival curves of WEHI 164-bearing mice treated with different doses of poly(I:C), daily, for 3 days, peritumorally, after 50% debulk of the primary tumor; n = 8–10 mice per group. Poly(I:C) doses: 1 μg/day (low dose [LD]), 10 μg/day (medium dose [MD]), or 50 μg/day (high dose [HD]).

(G and H) Survival curves of WEHI 164-bearing mice treated with poly(I:C) (10 μg/day), comparing 3 versus 6 days (G) or 6 versus 14 days (H); n = 8–10 mice per group.

(I and J) Survival curves of CT26-bearing mice (I) or AE17-bearing mice (J) treated with poly(I:C), i.t., daily, for 6 days; n = 8–10 mice per group. NS, not significant; Poly(I:C), polyinosinic:polycytidylic acid; DMXAA, 5,6-dimethylxantenone-4-acetic acid; rIFNβ, recombinant interferon beta; rIFNα1, recombinant interferon alpha. In (B) and (F)–(J), experiments were performed twice, and statistical analyses were performed using the log rank (Mantel-Cox) test to compare survival. Significance is represented using asterisks as follows: *$p \leq 0.05$, **$p \leq 0.005$, ***$p \leq 0.0005$, and ****$p \leq 0.0001$.

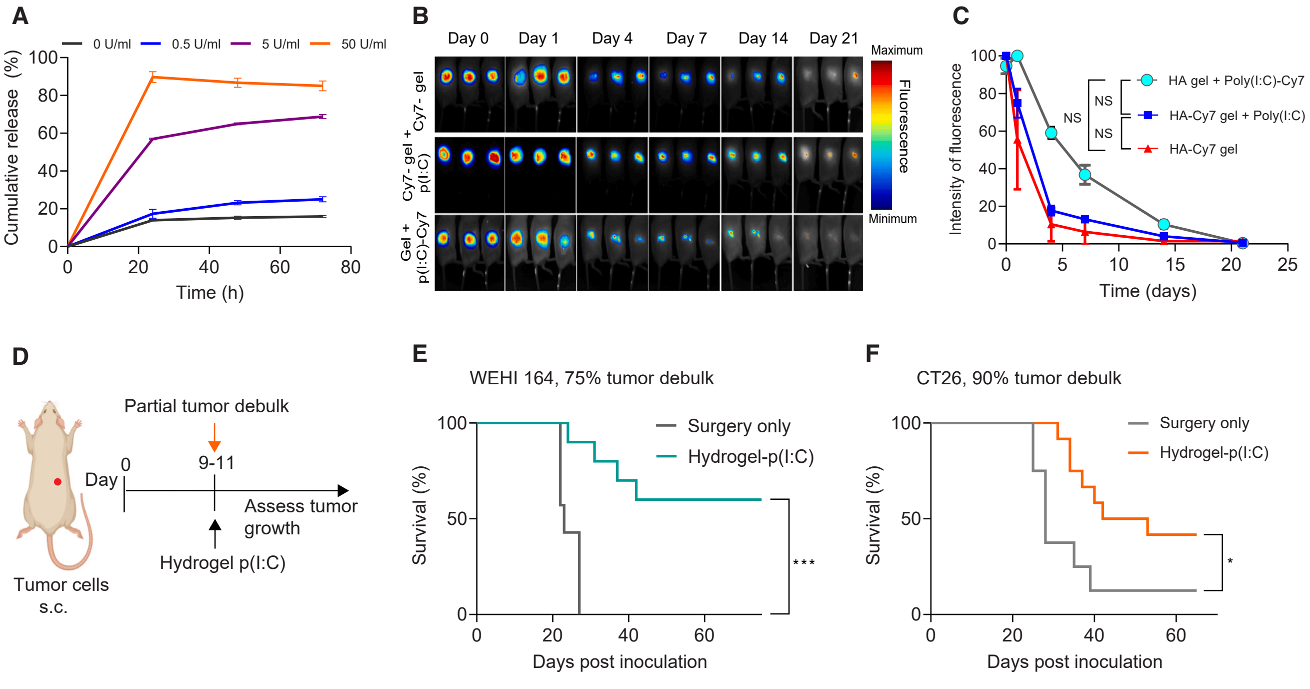

Figure 3. An intraoperative poly(I:C)-releasing hydrogel prevents tumor recurrence

(A) *In vitro* cumulative release profile of poly(I:C) from the optimized hydrogel with different concentration of hyaluronidase. Data are mean ± SD; n = 3 technical replicates. The experiment was performed once.

(B) Representative *in vivo* fluorescence images. Top row: Cy7-labeled gel, without poly(I:C), as a control arm. Middle row: Cy7-labeled gel plus unlabeled poly(I:C) as a control arm. Bottom row: Cy7-labeled poly(I:C) encapsulated in the hydrogel; n = 3 mice per group. The experiment was performed twice.

(C) *In vivo* poly(I:C)-Cy7 release profile. Cy7 signal as total signal ($\times 10^6$ phot/cm^2/s). Data are mean ± SD; n = 3 mice per group. Statistical comparison was made using one-way ANOVA followed by Tukey's multiple-comparison test. NS, not significant.

(D–F) Efficacy of hydrogel-poly(I:C) implanted in the tumor resection cavity after incomplete tumor resection. (D) Experimental design. A 75% debulk (WEHI 164 tumors) or 90% debulk (CT26 tumors) was performed and 100 μL of hydrogel-loaded poly(I:C) (250 μg) or empty hydrogel was placed in the resection cavity; n = 8–10 mice per group. The experiments were performed twice. (E and F) Survival curves of mice treated with hydrogel-poly(I:C) in WEHI 164 (E) or CT26 (F). In (E) and (F), statistical analyses were performed using the log rank (Mantel-Cox) test to compare survival. Significance is represented using asterisks as follows: *p ≤ 0.05, **p ≤ 0.005, ***p ≤ 0.0005, ****p ≤ 0.0001.

tumor recurrence following surgery in multiple cancer models, resulting in long-term systemic anti-cancer immunity.

Prolonged poly(I:C) administration induces a transient IFNα response, attracting inflammatory myeloid cells and depleting regulatory T cells in the TME

The main mechanism of action of poly(I:C) is thought to be type I IFN induction, in particular IFNβ.[33] However, the downstream immunological mechanisms for its anti-cancer effect are not completely understood. We therefore first assessed which immune cells take up poly(I:C) within the TME using fluorescein-labeled poly(I:C). We found that poly(I:C) was taken up predominately by macrophages (Figures 4A, 4B, and S3A). Next, we characterized the changes in the TME during prolonged poly(I:C) treatment using RNA sequencing (RNA-seq) at five time points in the WEHI 164 model (Figure 4C). A heatmap of the differentially expressed genes per sample across time demonstrated that the majority of genes were upregulated on day 3 and 5 in poly(I:C) treated tumors only (Figure 4D). Having observed these time-dependent differences between treated and untreated tumors, we employed time course sequencing (TC-seq) which clusters genes on the basis of their similarity in expression over time. We identified four clusters of genes that displayed differential expression patterns in time between treated and untreated tumors. Cluster 1 (Figure 4E) and cluster 2 (Figure 4F) contained genes that displayed a fast on/off activation pattern over the course of the six day poly(I:C) treatment. Pathway analysis identified that cluster 1 was enriched for genes involved in type I/II IFN signaling (Figure 4G) whereas cluster 2 was enriched for genes involved in T cell and natural killer (NK) cell activation (Figure 4H). Conversely, we observed that the two other clusters (cluster 3 and 4), which contained cancer-related genes, were downregulated over the course of the poly(I:C) treatment (Figures S3B–S3E).

Because it is not possible to computationally identify the type of IFN that is driving an observed gene expression signature, we performed functional experiments using blocking antibodies against IFNα, IFNβ, or the common IFNα/β receptor (IFNAR1).[34] Surprisingly, blocking IFNβ had no effect while blocking IFNα or IFNAR1 completely abolished the anti-tumor response following poly(I:C) treatment (Figures 4I and 4J).

To understand the dynamics of immune cell infiltration during poly(I:C) treatment, we performed cellular deconvolution analysis on the RNA-seq data.[35] This demonstrated that poly(I:C)

CellPress
OPEN ACCESS

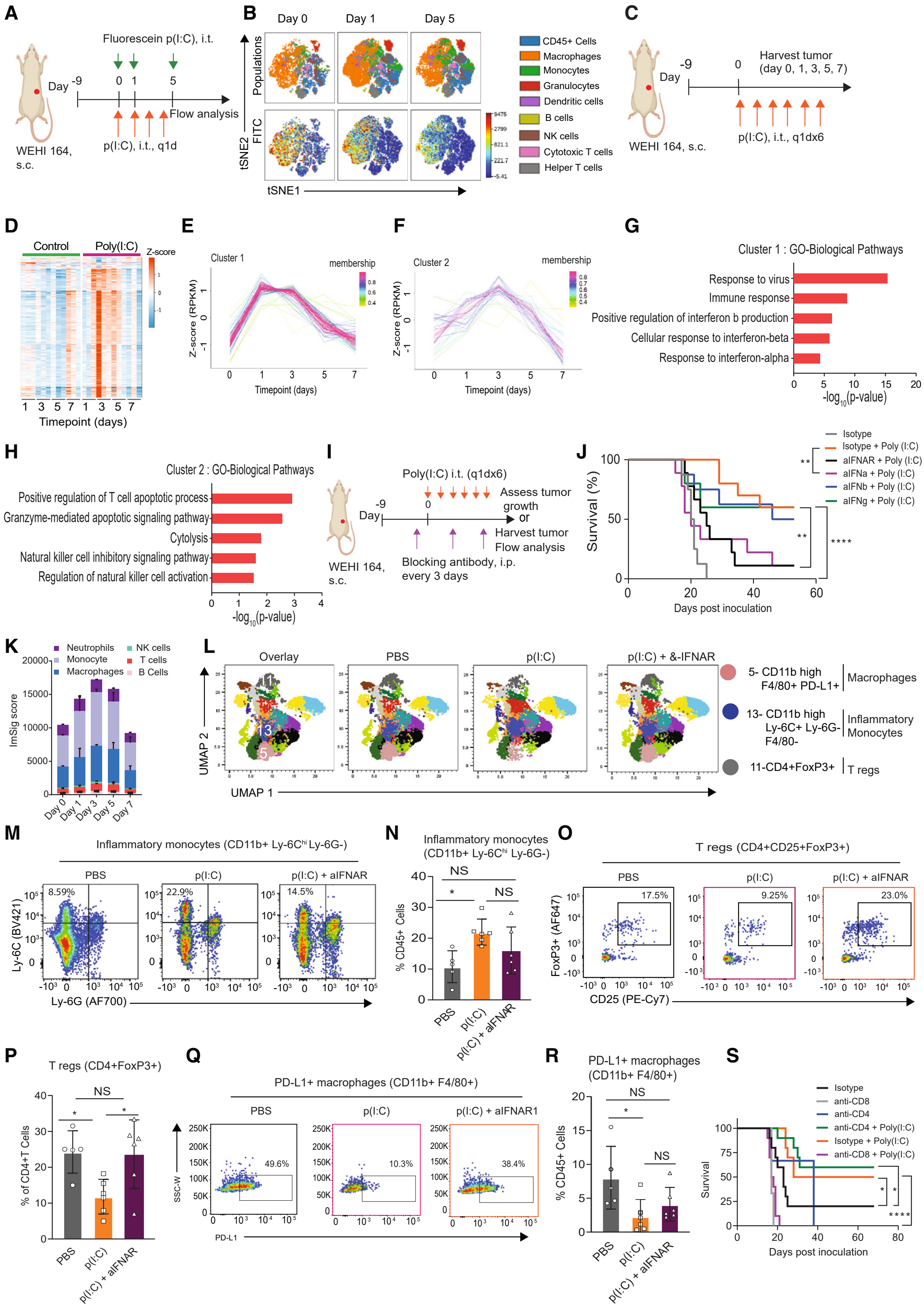

(legend on next page)

treatment resulted in a rapid but temporal immune cell infiltration dominated by myeloid cells, macrophages, and monocytes (Figure 4K).

To determine how poly(I:C)-induced IFNα modulates immune cell infiltration in the TME, we performed flow cytometry on WEHI 164 tumors following local poly(I:C) treatment in mice that were co-treated with or without an IFNAR1-blocking antibody. Local administration of poly(I:C) attracted inflammatory monocytes ($CD11b^{high}$ $Ly\text{-}6C^{+}$ $Ly\text{-}6G^{-}$), which were decreased when IFNAR1 was blocked at the same time (Figure 4L, cluster 13; Figures 4M and 4N). We analyzed gene expression levels of monocyte-attracting chemokines and found that they were indeed highly, yet transiently, upregulated in the poly(I:C) treated tumors, consistent with the transient monocyte infiltration after poly(I:C) treatment (Figure S3F). Poly(I:C) treatment also depleted T regs (Figure 4L, cluster 11; Figures 4O and 4P), and reduced the amount of PD-L1 expressing macrophages ($CD11b^{high}$ $F4/80^{+}$ $PD\text{-}L1^{+}$), both in an IFNAR1-dependent manner (Figure 4L, cluster 5; Figures 4Q, 4R, S3G–S3H, and S4).

To further understand which immune cells drive the response, we performed cell depletion experiments. Depletion of $CD4^{+}$ T helper cells did not change the response to i.t. poly(I:C), while depletion of $CD8^{+}$ cytotoxic T cell completely abrogated the anti-tumor response (Figure 4S).

Taken together, these data demonstrate that prolonged i.t. administration of poly(I:C) results in on/off IFNα activation, which reshapes the TME by attracting inflammatory myeloid cells and depleting regulatory T cells (Tregs) and that $CD8^{+}$ T cells are indispensable.

A pre-existing IFN gene signature predicts response to poly(I:C) hydrogel

Poly(I:C) is sensed mainly by TLR3 and the latter is not only expressed by tumor-infiltrating immune cells, but also by cancer cells themselves. Furthermore, immunogenic cell death of cancer cells has been reported following poly(I:C) treatment.[36] We therefore examined whether tumor-specific TLR3 expression was required for the observed anti-tumor effect of poly(I:C) by testing against TLR3 deficient WEHI 164 tumors. Although there was a slight decrease in the efficacy of poly(I:C) in tumors derived from TLR3 knockout (KO) cancer cells, this was not significant ($p = 0.08$; Figures 5A, 5B, S5A, and S5B), suggesting that TLR3 expression on tumor cells is not sufficient for the anti-cancer effect of poly(I:C).

As is frequent the case in immuno-oncology,[37] we noticed that the poly(I:C) hydrogel treatment resulted in dichotomous responses (Figures S5C). To identify the features of the TME predictive of response, we performed RNA-seq on the resected portion of tumor which was removed at the time of partial resection before the application of poly(I:C) hydrogel (Figure 5C). Cellular deconvolution analysis using CIBERSORT[38] showed that responsive tumors had higher levels of immune cell infiltration (Figure S5D), and gene set enrichment analysis (GSEA) showed that responsive tumors were enriched for genes associated with inflammatory pathways (IL-6, IL-2, and TNF-α signaling) and both type I and II IFN response (Figures 5D, S5E, and S5F).

Taken together, these data suggest that a pre-existing inflamed, IFN-activated TME is predictive of response to poly(I:C) hydrogel therapy.

The poly(I:C) hydrogel sensitizes tumors to PD-1 and CTLA-4 blockade

Given that we previously found that fast on/off activation of type I IFN underlies the response to immune checkpoint therapy (ICT),[24] we hypothesized that the poly(I:C)-releasing hydrogel could further amplify the ICT-induced anti-tumor response. Therefore, we assessed the effect of the poly(I:C) hydrogel in

Figure 4. Prolonged poly(I:C) administration induces a transient IFNα response attracting inflammatory myeloid cells and depleting Tregs in the TME

(A) Experimental setup for poly(I:C) uptake studies. Mice bearing WEHI 164 tumors were treated with poly(I:C), i.t., daily, for 4 days and a single injection of fluorescein-labeled poly(I:C) (50 μg), i.t., one hour before harvesting tumors for flow cytometry; n = 3 mice per group.
(B) Representative t-distributed stochastic neighbor embedding (t-SNE) plots.
(C–H) Time-dependent analysis of gene expression in poly(I:C)- or vehicle-treated tumors.
(C) Experimental design, and treatment strategy.
(D) Heatmap of differentially expressed genes between poly(I:C) and vehicle-treated groups across the different time points; n = 3 mice per group.
(E–H) TC-seq analysis was used to cluster genes with similar expression over time. (E and F) Gene expression over time for cluster 1 and cluster 2. (G and H) Top 5 upregulated biological pathways in clusters 1 and 2. In (A)–(H), the experiments were performed once.
(I) Experimental design for cytokine blocking studies; n = 8–10 mice per group. The experiments were performed twice.
(J) Survival curves of WEHI 164-bearing mice treated with poly(I:C) with or without blocking IFNα or IFNβ, or their receptor IFNAR, or blocking IFNγ. Statistical analyses were performed using the log rank (Mantel-Cox) test to compare survival. Significance is represented using asterisks as follows: *$p \leq 0.05$, **$p \leq 0.005$, ***$p \leq 0.0005$, ****$p \leq 0.0001$.
(K) Deconvolution analysis of RNA-seq data from Figures 1C and 1D; n = 3 mice per group.
(L–R) Mice bearing WEHI 164 tumors were treated with 2 doses of anti-IFNAR1 starting one day prior to 4 daily i.t. poly(I:C) injections. Tumors were harvested at day 4 for flow cytometry analysis; n = 5–6 mice per group. The experiments were performed twice. (L) UMAP showing clustering of cell populations across different treatment groups. (M and N) Representative FACS plots and proportion of inflammatory monocytes ($CD11b^{+}Ly\text{-}6C^{high}Ly\text{-}6G^{-}$). (O and P) Representative FACS plots and proportion of Tregs ($CD4^{+}FoxP3^{+}$). (Q and R) Representative FACS plots and proportion of $PD\text{-}L1^{+}$ macrophages ($CD11b^{high}F4/80^{+}PD\text{-}L1^{+}$). Data are mean ± SD; n = 5–6 biologically independent samples per group. In (L)–(R), statistical analyses were performed using one-way ANOVA followed by Tukey's multiple-comparison test. NS, not significant.
(S) Survival curves of WEHI 164-bearing mice treated with poly(I:C) with or without anti-CD4 or anti-CD8α cell depleting monoclonal antibodies; n = 8–10 mice per group. The experiments were performed twice. Statistical analyses were performed using the log rank (Mantel-Cox) test to compare survival. Significance is represented using asterisks as follows: *$p \leq 0.05$, **$p \leq 0.005$, ***$p \leq 0.0005$, ****$p \leq 0.0001$.
See also Figures S3, S4, and S6A.

CellPress
OPEN ACCESS

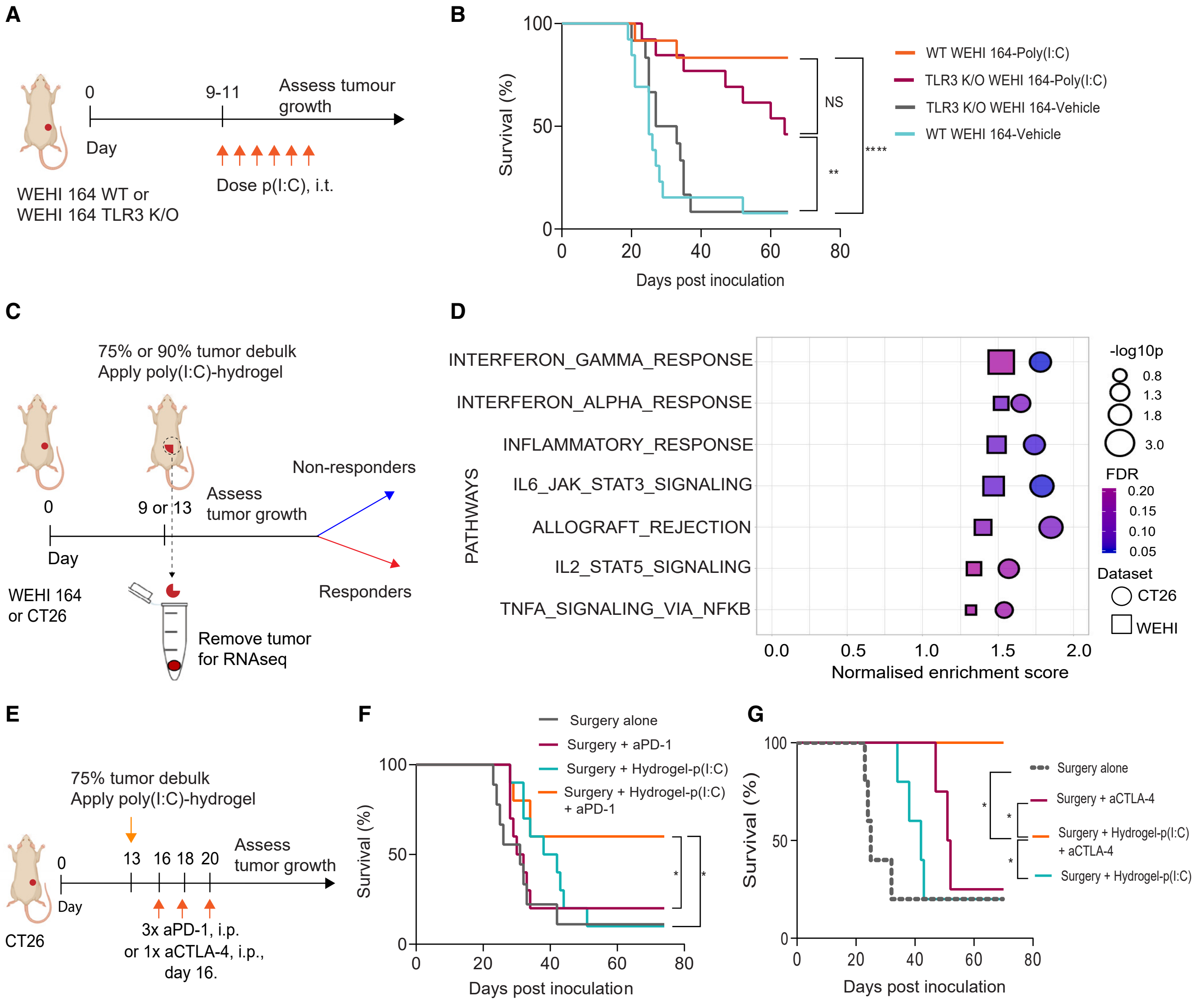

Figure 5. A pre-existing IFN gene signature predicts response to poly(I:C) hydrogel which sensitizes tumors to PD-1 and CTLA-4 blockade
(A and B) Efficacy of poly (I:C) in tumors derived from TLR3 KO WEHI 164 cells.
(A) Experimental design and treatment strategy.
(B) Survival curves of wild-type WEHI 164- or WEHI 164 TLR3 KO-bearing mice treated with poly(I:C); n = 8–10 mice per group. The experiments were performed twice. See also Figures S5A and S5B.
(C and D) RNA-seq for responders versus non-responders. (C) Experimental design, and treatment strategy. Partially resected tumors were kept in RNAlater for subsequent RNA extraction and sequencing. Treated mice were assigned to responder and non-responder groups depending on tumor outgrowth; n = 4–6 mice per group. The experiment was performed once in (D).
(D) GSEA top hallmark gene sets in responsive versus nonresponsive tumors. IL2, interleukin-2; IL6, interleukin-6; JAK, Janus kinase; STAT3/5, signal transducer and activator of transcription 3/5. See also Figures S5C–S5F.
(E–G) Combination of hydrogel poly(I:C) with anti-PD-1 or anti-CTLA-4. (E) Experimental design and treatment strategy. (F and G) Survival of CT26-bearing mice treated with poly(I:C) hydrogel in combination with anti-PD-1 (F) or anti-CTLA-4 (G); n = 8–10 mice per group. The experiments were performed twice.
In (B), (F), and (G), statistical analyses were performed using the log rank (Mantel-Cox) test to compare survival. Significance is represented using asterisks as follows: *$p \leq 0.05$, **$p \leq 0.005$, ***$p \leq 0.0005$, ****$p \leq 0.0001$.

combination with ICT, in incompletely resected CT26 tumors, leaving a large tumor bulk behind after surgery (25% instead of 10%, as previous). In this setting, the anti-PD-1 antibody, or the poly(I:C) hydrogel alone provided little benefit (Figure 5F). However, the poly(I:C) hydrogel in combination with anti-PD-1 antibody resulted in 60% of mice remaining free from tumor recurrence (p = 0.01; Figure 5F). This beneficial effect was also seen in combination with anti-CTLA-4 (Figure 5G). Together, these data show that intraoperative poly(I:C) hydrogel reshapes the TME, sensitizing tumors to ICT.

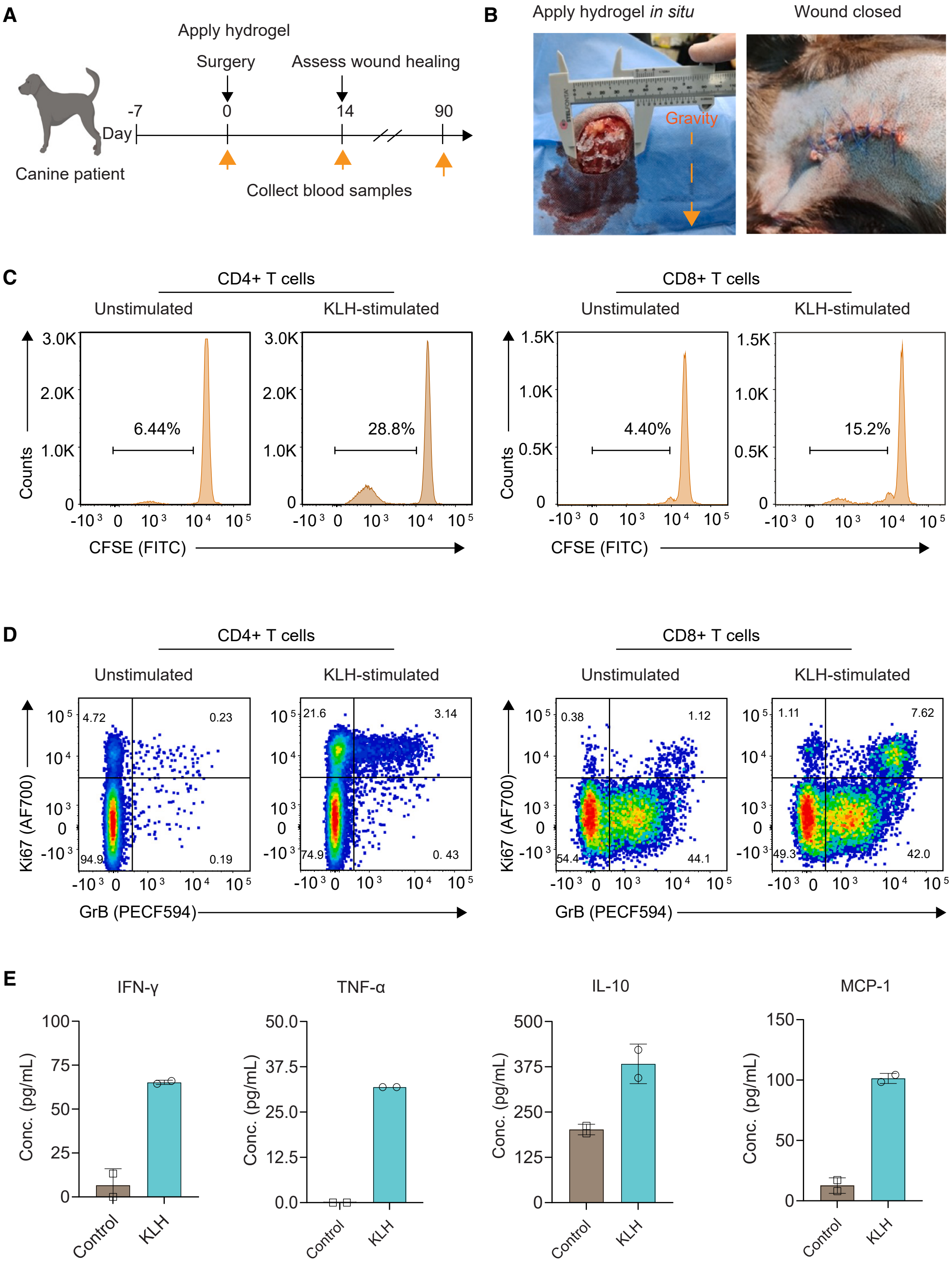

Figure 6. Safety, feasibility, and immunostimulatory potential of the poly(I:C) hydrogel in a canine trial

(A) Experimental design. Canine patients received intraoperative KLH/poly(I:C) hydrogel, containing 0.2 mg poly(I:C) and 1 mg KLH, at the time of surgery. Patient blood samples were taken directly prior to surgery, and at 2 weeks post-surgery. See STAR Methods for canine patient characteristics.

(B) Representative photographs of hydrogel application during surgical resection of a soft tissue tumor in a canine patient.

(legend continued on next page)

Safety, feasibility, and immunostimulatory potential of the poly(I:C) hydrogel in a canine trial

To assess the safety and surgical feasibility of delivering the poly(I:C) hydrogel during oncological surgery in a real-world setting of large surgical oncology wounds, we performed a veterinary clinical trial in canine soft tissue tumors. We incorporated the protein keyhole limpet hemocyanin (KLH) into the hydrogel, to act as a systemic biomarker of local antigen-specific immune activation.[39]

Following surgical removal of the soft tissue tumors, the hydrogel could be easily applied in the wound area and adhered well to the underlying tissue, without leakage after wound closure (Figure 6B; Video S6). There were no apparent side effects, and the surgical wounds healed well in all three patients, as assessed by follow-up examination 2 weeks post-surgery.

Following treatment, KLH-specific $CD4^+$ and $CD8^+$ T cell proliferation was measurable in peripheral blood upon restimulation with KLH (Figure 6C). Both $CD4^+$ and $CD8^+$ T cells expressed the proliferative marker Ki67, and $CD8^+$ T cells had elevated levels of granzyme B, indicating cytotoxic effector function (Figure 6D). Measurement of pro-inflammatory cytokines from KLH-stimulated cells showed increased production of inflammatory mediators including IFNγ, IL-6, TNF-α, and MCP-1 (Figure 6E). Taken together, these results show that intraoperative poly(I:C) immunotherapy, delivered via a hydrogel, is safe and surgically feasible in large surgical oncological wounds and can induce robust systemic T cell responses primed against a model antigen present in the wound/tumor microenvironment.

DISCUSSION

Cancer relapse after removal of the primary tumor remains a significant challenge. Combination treatment with surgery and immunotherapy provides a new therapeutic approach to prevent relapse and biomaterials provide a delivery tool for therapies during the intraoperative period. Previous studies have used biomaterials for targeted delivery of agonists of innate immune system,[7] adoptive T cell transfer[40] or combinations of chemotherapy and immunotherapy[8] in surgical settings. However, there were several important caveats to immunotherapy-releasing hydrogels that we addressed in this study.

First, hydrogels tested for drug delivery to date are either fluid, injectable, hydrogels,[41] or solid scaffolds,[7,40] and each of these formulations come with limitations. For local immunotherapy to work, it needs to remain at the site where it is applied. If a gel is not sufficiently solid, it may leak from the wound or dissipate away from the anatomical area where it was applied once a patient regains mobility after surgery. Conversely, if a gel is too solid, it cannot be easily applied in confined surgical spaces and may not adhere properly to the tissue. Taking these factors into account, our hydrogel was specifically optimized for application in surgical oncology.

Second, the wound healing response following large oncological surgery is a highly complex and orchestrated set of biological events that could easily be negatively affected by local therapies.[42] In addition, studies have shown that acute wound healing following oncological surgery can promote cancer relapse and metastasis.[43] We found that prolonged, low-dose i.t. poly(I:C) not only resulted in a strong therapeutic anti-cancer effect, but also did not affect wound healing negatively. The STING agonist DMXAA, in contrast, impaired wound healing, which is consistent with reports of patients with a gain-of-function mutation in STING having severe wound healing disorders.[44]

Third, although clinical studies have previously investigated i.t. poly(I:C) in humans,[45,46] this has always been through local injections, which require invasive procedures, thus significantly limiting the number of administrations that can be given to a patient. Given that for most immunotherapeutics the relationship between dose and response (and duration and response) is non-linear,[12] optimization of these variables is crucial. With our optimized schedule and dose, we found that poly(I:C) released from the gel induced a systemic memory response, corroborating previous results in melanoma patients treated with i.t. poly(I:C).[46]

Fourth, many cancers for which resection of the primary tumor is standard of care, are relatively resistant to ICT.[47–49] We found that our poly(I:C) hydrogel significantly increased the anti-tumor response of either anti-PD-1 or anti-CTLA-4 therapy. Mechanistically, we show that the therapeutic effect of poly(I:C) is dependent on temporarily restricted activation of IFNα, in line with previous results in ICT,[24] which likely explains the sensitizing effect to ICT by our poly(I:C) hydrogel. In addition, we show that a preexisting IFN gene expression signature is predictive of response to the poly(I:C) hydrogel. This is consistent with a positive feedforward loop between IFN activity and TLR3 expression, resulting in enhanced sensitivity to poly(I:C) in an IFN-rich microenvironment.[50,51] How this feedforward loop is halted during prolonged poly(I:C) treatment remains to be established. The fact that TLR3 deficient tumors were slightly less sensitive to poly(I:C), and that its beneficial effect could not be 100% negated by anti-IFNAR1 antibodies leaves room for a small role for non-IFN-dependent mechanisms of action, as has been shown recently for STING agonists.[52]

Finally, although mouse models have been crucial for the development of cancer immunotherapies,[53,54] they have relevant immunological differences compared with humans.[55] Canine studies represent an important bridge between mouse and human studies, where cancers develop slowly and spontaneously in genetically outbred animals in the presence of an intact immune system.[56] In addition, the human wound healing response is better represented in dogs than in mice.[57] It also allows a more faithful replication of oncological surgery as tumors in canine patients are of a relatively similar size to those in humans.[56] Testing the surgically optimized hydrogel in canine

(C and D) Representative FACS plots for KLH-specific proliferation (C) or Ki67 and granzyme B expression (D) in $CD4^+$ and $CD8^+$ T cells from canine patient peripheral blood mononuclear cells (PBMCs) collected 2 weeks post-surgery. PBMCs were restimulated in complete media with or without KLH (20 μg/mL) for 72 h prior to flow cytometry analysis. CFSE, carboxyfluorescein succinimidyl ester. See also Figure S6B.

(E) Quantification of pro-inflammatory cytokines in culture supernatants taken 48 h after *ex vivo* restimulation of PBMCs. Representative data are from one canine patient. Data are from one biological independent subject. In (E), two technical replicates were performed.

soft tissue tumor patients, we found it was easy to administer by the surgeon, adhered well to tissue and did not leak out of the closed wound. There was no interference with normal wound healing and importantly, we could detect a strong systemic T cell response against a model antigen that was present locally at the tumor/wound site.

Our results demonstrate that a poly(I:C)-releasing surgically optimized hydrogel is an effective and safe treatment to prevent cancer relapse after oncological surgery, warranting further translation into human trials.

Limitations of the study

Although subcutaneous tumor resection models are indispensable in assessing the efficacy of intraoperative therapies to prevent local cancer recurrence, these models do not give rise to distant metastases which are common in patients with cancer. Furthermore, these subcutaneous models do not necessarily recapitulate the surgical response in the diverse tissues of human cancers. The use of mouse surgery models incorporating orthotopic tumors and metastases could provide further evidence for the systemic effects of the locally applied gel.

It will be of interest to define the phenotype of the macrophages that take up the poly(I:C), which will be of value in the clinical translation of our findings.

Last, although the canine veterinary trial provides information about the safety and surgical utility of the poly(I:C) hydrogel in a real-world oncological setting, the limited sample size prevents drawing conclusions on the clinical activity. Expansion of the clinical trial cohort will provide more data.

STAR★METHODS

Detailed methods are provided in the online version of this paper and include the following:

- KEY RESOURCES TABLE
- RESOURCE AVAILABILITY
  - Lead contact
  - Materials availability
  - Data and code availability
- EXPERIMENTAL MODEL AND SUBJECT DETAILS
  - Mice
  - Canine subjects
  - Cell lines
- METHOD DETAILS
  - Preparation of hydrogels
  - Characterization of mechanical properties of hydrogel
  - *In vitro* release study
  - *In vivo* degradation study and drug-release profile
  - Surgical utility of hydrogels
  - Intratumoral therapy for subcutaneous model
  - *In vivo* wound healing model
  - Anti-tumor efficacy of hydrogel-poly(I:C) after incomplete tumor resection
  - *In vivo* ICT treatment
  - *In vivo* antibody blocking
  - *In vivo* cell depletion
  - *In vivo* cellular uptake of poly(I:C)
  - Flow cytometry staining and FACS analysis
  - RNA-seq extraction
  - Deletion of TLR3 by CRISPR/Cas9
  - Surgical feasibility in canine soft tissue cancer
  - Canine PBMC isolation
  - KLH T cell proliferation assay
  - Canine luminex multiplex cytokine assay
- QUANTIFICATION AND STATISTICAL ANALYSIS
  - Analysis of RNAseq data
  - Statistics and data analysis

SUPPLEMENTAL INFORMATION

Supplemental information can be found online at https://doi.org/10.1016/j.xcrm.2023.101113.

ACKNOWLEDGMENTS

F.X.R. and B.V. are supported by Australian Government PhD scholarships and Abbie Basson PhD top-up scholarships. B.W. is supported by a Cancer Council WA Fellowship. R.Z. is supported by a Forrest Fellowship and a Raine Priming Grant. W.J.L. was supported by fellowships from the Simon Lee Foundation and the National Health and Medical Research Council (NHMRC). This work was funded by grant APP1184331 from Cancer Australia/The Kids' Cancer Project and supported by funding from the Sock it to Sarcoma! Foundation, the Australian and New Zealand Sarcoma Association, Perpetual, and the Children's Leukaemia and Cancer Research Foundation. The authors acknowledge the facilities and the scientific and technical assistance offered by Diana Patalwala at the National Imaging Facility at the Centre for Microscopy, Characterization & Analysis, The University of Western Australia, a facility funded by the University, State, and Commonwealth Governments. Icons of mice and dogs were created with BioRender.com.

AUTHOR CONTRIBUTIONS

Conceptualization, K.S.I. and W.J.L.; methodology, F.X.R., C.W.E., B.W., M.N., M.S.H., B.F.K., R.M.Z., K.S.I., and W.J.L.; formal analysis, F.X.R., C.W.E., B.W., M.S.H., R.M.Z., and V.S.F.; investigation, F.X.R., C.W.E., B.W., M.N., B.V., D.H., D.N., E.A.R., T.W., M.S.H., R.W.S., V.S.F., C.A.F., K.W., and R.M.Z.; resources, M.N. and K.W.; writing – original draft, F.X.R., C.W.E., B.W., K.S.I., and W.J.L.; writing – review & editing, all authors; visualization, F.X.R., B.W., R.M.Z., and W.J.L.; supervision, B.W., R.M.Z., and K.S.I., and W.J.L.; project administration, F.X.R., B.W., K.S.I., and W.J.L.; funding acquisition, K.S.I. and W.J.L.

DECLARATION OF INTERESTS

We declare a patent application pertaining to aspects of this work. B.F.K. discloses a financial interest in OncoRes Medical, a company developing QME for use in surgery. W.J.L. declares consultancy for Douglas Pharmaceuticals and MSD and research funding from Douglas Pharmaceuticals, AstraZeneca, and ENA Therapeutics.

INCLUSION AND DIVERSITY

We support inclusive, diverse, and equitable conduct of research.

Received: December 14, 2022
Revised: May 10, 2023
Accepted: June 18, 2023
Published: July 18, 2023

CellPress
OPEN ACCESS

REFERENCES

1. Wyld, L., Audisio, R.A., and Poston, G.J. (2015). The evolution of cancer surgery and future perspectives. Nat. Rev. Clin. Oncol. *12*, 115–124. https://doi.org/10.1038/nrclinonc.2014.191.
2. Mahvi, D.A., Liu, R., Grinstaff, M.W., Colson, Y.L., and Raut, C.P. (2018). Local cancer recurrence: The realities, challenges, and opportunities for new therapies. CA A Cancer J. Clin. *68*, 488–505. https://doi.org/10.3322/caac.21498.
3. Eggermont, A.M.M., Blank, C.U., Mandala, M., Long, G.V., Atkinson, V., Dalle, S., Haydon, A., Lichinitser, M., Khattak, A., Carlino, M.S., et al. (2018). Adjuvant pembrolizumab versus placebo in resected stage iii melanoma. N. Engl. J. Med. *378*, 1789–1801. https://doi.org/10.1056/NEJMoa1802357.
4. Forde, P.M., Chaft, J.E., Smith, K.N., Anagnostou, V., Cottrell, T.R., Hellmann, M.D., Zahurak, M., Yang, S.C., Jones, D.R., Broderick, S., et al. (2018). Neoadjuvant pd-1 blockade in resectable lung cancer. N. Engl. J. Med. *378*, 1976–1986. https://doi.org/10.1056/NEJMoa1716078.
5. Cloughesy, T.F., Mochizuki, A.Y., Orpilla, J.R., Hugo, W., Lee, A.H., Davidson, T.B., Wang, A.C., Ellingson, B.M., Rytlewski, J.A., Sanders, C.M., et al. (2019). Neoadjuvant anti-pd-1 immunotherapy promotes a survival benefit with intratumoral and systemic immune responses in recurrent glioblastoma. Nat. Med. *25*, 477–486. https://doi.org/10.1038/s41591-018-0337-7.
6. Weber, J., Mandala, M., Del Vecchio, M., Gogas, H.J., Arance, A.M., Cowey, C.L., Dalle, S., Schenker, M., Chiarion-Sileni, V., Marquez-Rodas, I., et al. (2017). Adjuvant nivolumab versus ipilimumab in resected stage iii or iv melanoma. N. Engl. J. Med. *377*, 1824–1835. https://doi.org/10.1056/NEJMoa1709030.
7. Park, C.G., Hartl, C.A., Schmid, D., Carmona, E.M., Kim, H.J., and Goldberg, M.S. (2018). Extended release of perioperative immunotherapy prevents tumor recurrence and eliminates metastases. Sci. Transl. Med. *10*, eaar1916. https://doi.org/10.1126/scitranslmed.aar1916.
8. Wang, C., Wang, J., Zhang, X., Yu, S., Wen, D., Hu, Q., Ye, Y., Bomba, H., Hu, X., Liu, Z., et al. (2018). In situ formed reactive oxygen species-responsive scaffold with gemcitabine and checkpoint inhibitor for combination therapy. Sci. Transl. Med. *10*, eaan3682. https://doi.org/10.1126/scitranslmed.aan3682.
9. Wang, H., and Mooney, D.J. (2018). Biomaterial-assisted targeted modulation of immune cells in cancer treatment. Nat. Mater. *17*, 761–772. https://doi.org/10.1038/s41563-018-0147-9.
10. Li, J., and Mooney, D.J. (2016). Designing hydrogels for controlled drug delivery. Nat. Rev. Mater. *1*, 16071. https://doi.org/10.1038/natrevmats.2016.71.
11. Wang, H., Najibi, A.J., Sobral, M.C., Seo, B.R., Lee, J.Y., Wu, D., Li, A.W., Verbeke, C.S., and Mooney, D.J. (2020). Biomaterial-based scaffold for in situ chemo-immunotherapy to treat poorly immunogenic tumors. Nat. Commun. *11*, 5696. https://doi.org/10.1038/s41467-020-19540-z.
12. Lesterhuis, W.J., Haanen, J.B.A.G., and Punt, C.J.A. (2011). Cancer immunotherapy – revisited. Nat. Rev. Drug Discov. *10*, 591–600. https://doi.org/10.1038/nrd3500.
13. Fakhari, A., and Berkland, C. (2013). Applications and emerging trends of hyaluronic acid in tissue engineering, as a dermal filler and in osteoarthritis treatment. Acta Biomater. *9*, 7081–7092. https://doi.org/10.1016/j.actbio.2013.03.005.
14. Park, S.H., Seo, J.Y., Park, J.Y., Ji, Y.B., Kim, K., Choi, H.S., Choi, S., Kim, J.H., Min, B.H., and Kim, M.S. (2019). An injectable, click-crosslinked, cytomodulin-modified hyaluronic acid hydrogel for cartilage tissue engineering. NPG Asia Mater. *11*, 30. https://doi.org/10.1038/s41427-019-0130-1.
15. Rohrich, R.J., Ghavami, A., and Crosby, M.A. (2007). The role of hyaluronic acid fillers (restylane) in facial cosmetic surgery: Review and technical considerations. Plast. Reconstr. Surg. *120*, 41S–54S. https://doi.org/10.1097/01.prs.0000248794.63898.0f.
16. Nyman, E., Henricson, J., Ghafouri, B., Anderson, C.D., and Kratz, G. (2019). Hyaluronic acid accelerates re-epithelialization and alters protein expression in a human wound model. Plast. Reconstr. Surg. Glob. Open *7*, e2221. https://doi.org/10.1097/GOX.0000000000002221.
17. Humbert, P., Mikosinki, J., Benchikhi, H., and Allaert, F.A. (2013). Efficacy and safety of a gauze pad containing hyaluronic acid in treatment of leg ulcers of venous or mixed origin: A double-blind, randomised, controlled trial. Int. Wound J. *10*, 159–166. https://doi.org/10.1111/j.1742-481X.2012.00957.x.
18. Lee, J.H., Jung, J.Y., and Bang, D. (2008). The efficacy of topical 0.2% hyaluronic acid gel on recurrent oral ulcers: Comparison between recurrent aphthous ulcers and the oral ulcers of behcet's disease. J. Eur. Acad. Dermatol. Venereol. *22*, 590–595. https://doi.org/10.1111/j.1468-3083.2007.02564.x.
19. Shu, X.Z., Liu, Y., Luo, Y., Roberts, M.C., and Prestwich, G.D. (2002). Disulfide cross-linked hyaluronan hydrogels. Biomacromolecules *3*, 1304–1311. https://doi.org/10.1021/bm025603c.
20. Kennedy, K.M., Chin, L., McLaughlin, R.A., Latham, B., Saunders, C.M., Sampson, D.D., and Kennedy, B.F. (2015). Quantitative micro-elastography: Imaging of tissue elasticity using compression optical coherence elastography. Sci. Rep. *5*, 15538. https://doi.org/10.1038/srep15538.
21. Bulpitt, P., and Aeschlimann, D. (1999). New strategy for chemical modification of hyaluronic acid: Preparation of functionalized derivatives and their use in the formation of novel biocompatible hydrogels. J. Biomed. Mater. Res. *47*, 152–169. https://doi.org/10.1002/(sici)1097-4636(199911)47:2<152::Aid-jbm5>3.0.Co;2-i.
22. Di Domizio, J., Belkhodja, C., Chenuet, P., Fries, A., Murray, T., Mondéjar, P.M., Demaria, O., Conrad, C., Homey, B., Werner, S., et al. (2020). The commensal skin microbiota triggers type i ifn-dependent innate repair responses in injured skin. Nat. Immunol. *21*, 1034–1045. https://doi.org/10.1038/s41590-020-0721-6.
23. Zemek, R.M., De Jong, E., Chin, W.L., Schuster, I.S., Fear, V.S., Casey, T.H., Forbes, C., Dart, S.J., Leslie, C., Zaitouny, A., et al. (2019). Sensitization to immune checkpoint blockade through activation of a stat1/nk axis in the tumor microenvironment. Sci. Transl. Med. *11*, eaav7816. https://doi.org/10.1126/scitranslmed.aav7816.
24. Zemek, R.M., Chin, W.L., Fear, V.S., Wylie, B., Casey, T.H., Forbes, C., Tilsed, C.M., Boon, L., Guo, B.B., Bosco, A., et al. (2022). Temporally restricted activation of ifnβ signaling underlies response to immune checkpoint therapy in mice. Nat. Commun. *13*, 4895. https://doi.org/10.1038/s41467-022-32567-8.
25. Talmadge, J.E., Adams, J., Phillips, H., Collins, M., Lenz, B., Schneider, M., and Chirigos, M. (1985). Immunotherapeutic potential in murine tumor models of polyinosinic-polycytidylic acid and poly-l-lysine solubilized by carboxymethylcellulose. Cancer Res. *45*, 1066–1072.
26. Talmadge, J.E., and Hartmann, D. (1985). Optimization of an Immunotherapeutic Protocol with poly(i,c)-lc. J. Biol. Response Modif. *4*, 484–489.
27. Black, P.L., Hartmann, D., Pennington, R., Phillips, H., Schneider, M., Tribble, H.R., and Talmadge, J.E. (1992). Effect of tumor burden and route of administration on the immunotherapeutic properties of polyinosinic-polycytidylic acid stabilized with poly-l-lysine in carboxymethyl cellulose [poly(i,c)-lc]. Int. J. Immunopharm. *14*, 1341–1353. https://doi.org/10.1016/0192-0561(92)90005-6.
28. Corrales, L., Glickman, L.H., McWhirter, S.M., Kanne, D.B., Sivick, K.E., Katibah, G.E., Woo, S.R., Lemmens, E., Banda, T., Leong, J.J., et al. (2015). Direct activation of sting in the tumor microenvironment leads to potent and systemic tumor regression and immunity. Cell Rep. *11*, 1018–1030. https://doi.org/10.1016/j.celrep.2015.04.031.
29. Lesinski, G.B., Anghelina, M., Zimmerer, J., Bakalakos, T., Badgwell, B., Parihar, R., Hu, Y., Becknell, B., Abood, G., Chaudhury, A.R., et al. (2003). The antitumor effects of ifn-alpha are abrogated in a stat1-deficient mouse. J. Clin. Invest. *112*, 170–180. https://doi.org/10.1172/JCI16603.
30. Rwandamuriye, F.X., Weston, B.J., Johns, T.G., Lesterhuis, W.J., and Zemek, R.M. (2020). A mouse model of incompletely resected soft tissue

sarcoma for testing (neo)adjuvant therapies. J. Vis. Exp.e60882 https://doi.org/10.3791/60882.

31. Monzón, M.E., Manzanares, D., Schmid, N., Casalino-Matsuda, S.M., and Forteza, R.M. (2008). Hyaluronidase expression and activity is regulated by pro-inflammatory cytokines in human airway epithelial cells. Am. J. Respir. Cell Mol. Biol. *39*, 289–295. https://doi.org/10.1165/rcmb.2007-0361OC.

32. Toole, B.P. (2004). Hyaluronan: From extracellular glue to pericellular cue. Nat. Rev. Cancer *4*, 528–539. https://doi.org/10.1038/nrc1391.

33. Honda, K., Sakaguchi, S., Nakajima, C., Watanabe, A., Yanai, H., Matsumoto, M., Ohteki, T., Kaisho, T., Takaoka, A., Akira, S., et al. (2003). Selective contribution of ifn-alpha/beta signaling to the maturation of dendritic cells induced by double-stranded rna or viral infection. Proc. Natl. Acad. Sci. USA *100*, 10872–10877. https://doi.org/10.1073/pnas.1934678100.

34. Ng, C.T., Sullivan, B.M., Teijaro, J.R., Lee, A.M., Welch, M., Rice, S., Sheehan, K.C.F., Schreiber, R.D., and Oldstone, M.B.A. (2015). Blockade of interferon beta, but not interferon alpha, signaling controls persistent viral infection. Cell Host Microbe *17*, 653–661. https://doi.org/10.1016/j.chom.2015.04.005.

35. Nirmal, A.J., Regan, T., Shih, B.B., Hume, D.A., Sims, A.H., and Freeman, T.C. (2018). Immune cell gene signatures for profiling the microenvironment of solid tumors. Cancer Immunol. Res. *6*, 1388–1400. https://doi.org/10.1158/2326-6066.CIR-18-0342.

36. Aznar, M.A., Planelles, L., Perez-Olivares, M., Molina, C., Garasa, S., Etxeberría, I., Perez, G., Rodriguez, I., Bolaños, E., Lopez-Casas, P., et al. (2019). Immunotherapeutic effects of intratumoral nanoplexed poly i:C. J. ImmunoTher. Cancer *7*, 116. https://doi.org/10.1186/s40425-019-0568-2.

37. Lesterhuis, W.J., Bosco, A., Millward, M.J., Small, M., Nowak, A.K., and Lake, R.A. (2017). Dynamic versus static biomarkers in cancer immune checkpoint blockade: Unravelling complexity. Nat. Rev. Drug Discov. *16*, 264–272. https://doi.org/10.1038/nrd.2016.233.

38. Newman, A.M., Liu, C.L., Green, M.R., Gentles, A.J., Feng, W., Xu, Y., Hoang, C.D., Diehn, M., and Alizadeh, A.A. (2015). Robust enumeration of cell subsets from tissue expression profiles. Nat. Methods *12*, 453–457. https://doi.org/10.1038/nmeth.3337.

39. Lesterhuis, W.J., de Vries, I.J.M., Schreibelt, G., Lambeck, A.J.A., Aarntzen, E.H.J.G., Jacobs, J.F.M., Scharenborg, N.M., van de Rakt, M.W.M.M., de Boer, A.J., Croockewit, S., et al. (2011). Route of administration modulates the induction of dendritic cell vaccine-induced antigen-specific t cells in advanced melanoma patients. Clin. Cancer Res. *17*, 5725–5735. https://doi.org/10.1158/1078-0432.CCR-11-1261.

40. Stephan, S.B., Taber, A.M., Jileaeva, I., Pegues, E.P., Sentman, C.L., and Stephan, M.T. (2015). Biopolymer implants enhance the efficacy of adoptive t-cell therapy. Nat. Biotechnol. *33*, 97–101. https://doi.org/10.1038/nbt.3104.

41. Song, C., Phuengkham, H., Kim, Y.S., Dinh, V.V., Lee, I., Shin, I.W., Shin, H.S., Jin, S.M., Um, S.H., Lee, H., et al. (2019). Syringeable immunotherapeutic nanogel reshapes tumor microenvironment and prevents tumor metastasis and recurrence. Nat. Commun. *10*, 3745. https://doi.org/10.1038/s41467-019-11730-8.

42. Payne, W.G., Naidu, D.K., Wheeler, C.K., Barkoe, D., Mentis, M., Salas, R.E., Smith, D.J., Jr., and Robson, M.C. (2008). Wound healing in patients with cancer. Eplasty *8*, e9.

43. Krall, J.A., Reinhardt, F., Mercury, O.A., Pattabiraman, D.R., Brooks, M.W., Dougan, M., Lambert, A.W., Bierie, B., Ploegh, H.L., Dougan, S.K., and Weinberg, R.A. (2018). The systemic response to surgery triggers the outgrowth of distant immune-controlled tumors in mouse models of dormancy. Sci. Transl. Med. *10*, eaan3464. https://doi.org/10.1126/scitranslmed.aan3464.

44. Liu, Y., Jesus, A.A., Marrero, B., Yang, D., Ramsey, S.E., Sanchez, G.A.M., Tenbrock, K., Wittkowski, H., Jones, O.Y., Kuehn, H.S., et al. (2014). Activated sting in a vascular and pulmonary syndrome. N. Engl. J. Med. *371*, 507–518. https://doi.org/10.1056/NEJMoa1312625.

45. Salazar, A.M., Erlich, R.B., Mark, A., Bhardwaj, N., and Herberman, R.B. (2014). Therapeutic in situ autovaccination against solid cancers with intratumoral poly-iclc: Case report, hypothesis, and clinical trial. Cancer Immunol. Res. *2*, 720–724. https://doi.org/10.1158/2326-6066.CIR-14-0024.

46. Márquez-Rodas, I., Longo, F., Rodriguez-Ruiz, M.E., Calles, A., Ponce, S., Jove, M., Rubio-Viqueira, B., Perez-Gracia, J.L., Gómez-Rueda, A., López-Tarruella, S., et al. (2020). Intratumoral nanoplexed poly i:C bo-112 in combination with systemic anti-pd-1 for patients with anti-pd-1-refractory tumors. Sci. Transl. Med. *12*, eabb0391. https://doi.org/10.1126/scitranslmed.abb0391.

47. D'Angelo, S.P., Mahoney, M.R., Van Tine, B.A., Atkins, J., Milhem, M.M., Jahagirdar, B.N., Antonescu, C.R., Horvath, E., Tap, W.D., Schwartz, G.K., and Streicher, H. (2018). Nivolumab with or without ipilimumab treatment for metastatic sarcoma (alliance a091401): Two open-label, non-comparative, randomised, phase 2 trials. Lancet Oncol. *19*, 416–426. https://doi.org/10.1016/s1470-2045(18)30006-8.

48. Yarchoan, M., Hopkins, A., and Jaffee, E.M. (2017). Tumor mutational burden and response rate to pd-1 inhibition. N. Engl. J. Med. *377*, 2500–2501. https://doi.org/10.1056/NEJMc1713444.

49. O'Reilly, E.M., Oh, D.Y., Dhani, N., Renouf, D.J., Lee, M.A., Sun, W., Fisher, G., Hezel, A., Chang, S.C., Vlahovic, G., et al. (2019). Durvalumab with or without tremelimumab for patients with metastatic pancreatic ductal adenocarcinoma: A phase 2 randomized clinical trial. JAMA Oncol. *5*, 1431–1438. https://doi.org/10.1001/jamaoncol.2019.1588.

50. Tissari, J., Sirén, J., Meri, S., Julkunen, I., and Matikainen, S. (2005). Ifn-alpha enhances tlr3-mediated antiviral cytokine expression in human endothelial and epithelial cells by up-regulating tlr3 expression. J. Immunol. *174*, 4289–4294. https://doi.org/10.4049/jimmunol.174.7.4289.

51. Zhang, L., Xiang, W., Wang, G., Yan, Z., Zhu, Z., Guo, Z., Sengupta, R., Chen, A.F., Loughran, P.A., Lu, B., et al. (2016). Interferon beta (ifn-beta) production during the double-stranded rna (dsrna) response in hepatocytes involves coordinated and feedforward signaling through toll-like receptor 3 (tlr3), rna-dependent protein kinase (pkr), inducible nitric oxide synthase (inos), and src protein. J. Biol. Chem. *291*, 15093–15107. https://doi.org/10.1074/jbc.M116.717942.

52. Wu, J., Dobbs, N., Yang, K., and Yan, N. (2020). Interferon-independent activities of mammalian sting mediate antiviral response and tumor immune evasion. Immunity *53*, 115–126.e5. https://doi.org/10.1016/j.immuni.2020.06.009.

53. Leach, D.R., Krummel, M.F., and Allison, J.P. (1996). Enhancement of antitumor immunity by ctla-4 blockade. Science *271*, 1734–1736. https://doi.org/10.1126/science.271.5256.1734.

54. Iwai, Y., Ishida, M., Tanaka, Y., Okazaki, T., Honjo, T., and Minato, N. (2002). Involvement of pd-l1 on tumor cells in the escape from host immune system and tumor immunotherapy by pd-l1 blockade. Proc. Natl. Acad. Sci. USA *99*, 12293–12297. https://doi.org/10.1073/pnas.192461099.

55. Medetgul-Ernar, K., and Davis, M.M. (2022). Standing on the shoulders of mice. Immunity *55*, 1343–1353. https://doi.org/10.1016/j.immuni.2022.07.008.

56. Paoloni, M., and Khanna, C. (2008). Translation of new cancer treatments from pet dogs to humans. Nat. Rev. Cancer *8*, 147–156. https://doi.org/10.1038/nrc2273.

57. Lux, C.N. (2022). Wound healing in animals: A review of physiology and clinical evaluation. Vet. Dermatol. *33*, 91–e27. https://doi.org/10.1111/vde.13032.

58. Andrews, S. (2010). Fastqc: A quality control tool for high throughput sequence data. http://www.bioinformatics.babraham.ac.uk/projects/fastqc/.

59. Bray, N.L., Pimentel, H., Melsted, P., and Pachter, L. (2016). Near-optimal probabilistic rna-seq quantification. Nat. Biotechnol. *34*, 525–527. https://doi.org/10.1038/nbt.3519.

CellPress
OPEN ACCESS

60. Love, M.I., Huber, W., and Anders, S. (2014). Moderated estimation of fold change and dispersion for rna-seq data with deseq2. Genome Biol. *15*, 550. https://doi.org/10.1186/s13059-014-0550-8.

61. Subramanian, A., Tamayo, P., Mootha, V.K., Mukherjee, S., Ebert, B.L., Gillette, M.A., Paulovich, A., Pomeroy, S.L., Golub, T.R., Lander, E.S., and Mesirov, J.P. (2005). Gene set enrichment analysis: A knowledge-based approach for interpreting genome-wide expression profiles. Proc. Natl. Acad. Sci. USA *102*, 15545–15550. https://doi.org/10.1073/pnas.0506580102.

62. Wickham, H. (2016). Ggplot2: Elegant Graphics for Data Analysis, 1. Aufl. Edition (Springer-Verlag). https://doi.org/10.1007/978-0-387-98141-3.

63. Sanderson, R.W., Fang, Q., Curatolo, A., Adams, W., Lakhiani, D.D., Ismail, H.M., Foo, K.Y., Dessauvagie, B.F., Latham, B., Yeomans, C., et al. (2020). Camera-based optical palpation. Sci. Rep. *10*, 15951. https://doi.org/10.1038/s41598-020-72603-5.

64. Lesterhuis, W.J., Salmons, J., Nowak, A.K., Rozali, E.N., Khong, A., Dick, I.M., Harken, J.A., Robinson, B.W., and Lake, R.A. (2013). Synergistic effect of ctla-4 blockade and cancer chemotherapy in the induction of anti-tumor immunity. PLoS One *8*, e61895. https://doi.org/10.1371/journal.pone.0061895.

65. Fear, V.S., Forbes, C.A., Anderson, D., Rauschert, S., Syn, G., Shaw, N., Jones, M.E., Forrest, A.R., Baynam, G., and Lassmann, T. (2022). Functional validation of variants of unknown significance using crispr gene editing and transcriptomics: A kleefstra syndrome case study. Gene *821*, 146287. https://doi.org/10.1016/j.gene.2022.146287.

66. Pantelyushin, S., Ranninger, E., Bettschart-Wolfensberger, R., and Vom Berg, J. (2020). Omip-065: Dog immunophenotyping and t-cell activity evaluation with a 14-color panel. Cytometry A. *97*, 1024–1027. https://doi.org/10.1002/cyto.a.24168.

67. Futschik, M.E., and Carlisle, B. (2005). Noise-robust soft clustering of gene expression time-course data. J. Bioinf. Comput. Biol. *3*, 965–988. https://doi.org/10.1142/s0219720005001375.

68. Wu, M., and Gu, L. (2021). Tcseq: Time course sequencing data analysis. R package version 1.18.0. https://www.bioconductor.org/packages/release/bioc/html/TCseq.html.

69. Liberzon, A., Subramanian, A., Pinchback, R., Thorvaldsdóttir, H., Tamayo, P., and Mesirov, J.P. (2011). Molecular signatures database (msigdb) 3.0. Bioinformatics *27*, 1739–1740. https://doi.org/10.1093/bioinformatics/btr260.

STAR★METHODS

KEY RESOURCES TABLE

REAGENT or RESOURCE	SOURCE	IDENTIFIER
Antibodies		
Anti-mouse CD45⁻ BUV395 (clone 30-F11)	BD Biosciences	Cat: 564279; RRID: AB_2651134
Anti-mouse CD4- BUV496 (clone GK1.5)	BD Biosciences	Cat: 564667; RRID: AB_2722549
Anti-mouse CD3- BUV737 (clone 17A2)	BD Biosciences	Cat: 564380; RRID: AB_2738781
Anti-mouse Ly6C- BV421 (clone AL-21)	BD Biosciences	Cat: 562727; RRID: AB_2737748
Anti-mouse CD8- BV480 (clone 53-6.7)	BD Biosciences	Cat: 566096; RRID: AB_2739500
Anti-mouse MHCII- BV605 (clone M5/114.15.2)	BD Biosciences	Cat: 563413; RRID: AB_2738190
Anti-mouse CD19- BV650 (clone 1D3)	BD Biosciences	Cat: 563235; RRID: AB_2738085
Anti-mouse CD11c- BV711(clone HL3)	BD Biosciences	Cat: 563048; RRID: AB_2734778
Anti-mouse CD335- BV786 (clone 29A1.4)	BD Biosciences	Cat: 741029; RRID: AB_2740647
Anti-mouse Foxp3- AF488 (clone MF23)	BD Biosciences	Cat: 560403; RRID: AB_1645192
Anti-mouse F4/80- PerCP-Cy5.5 (clone T45-2342)	BD Biosciences	Cat: 746070; RRID: AB_2743450
Anti-mouse CD86- PE (clone GL1)	BD Biosciences	Cat: 553692; RRID: AB_394994
Anti-mouse CD11b- PE-Cy7 (clone M1/70)	BD Biosciences	Cat: 552850; RRID: AB_394491
Anti-mouse FOXP3- AF647 (clone MF23)	BD Biosciences	Cat: 560402; RRID: AB_1645202
Anti-mouse PD-L1-APC (clone MIH5)	BD Biosciences	Cat: 564715; RRID: AB_2687479
Anti-mouse Ly6G- AF700 (clone 1A8)	BD Biosciences	Cat: 561236; RRID: AB_10611860
Anti-mouse CD19- PerCP-Cy5.5 (clone 1D3)	BD Biosciences	Cat: 551001; RRID: AB_394004
Anti-mouse CD25- PE-Cy7 (clone PC61)	BD Biosciences	Cat: 561780; RRID: AB_10893596
Fixable Viability Stain 780	BD Biosciences	Cat: 565388; RRID: AB_2869673
Anti-dog CD3-FITC (clone CA17.2A12)	Bio-Rad	Cat: MCA1774F; RRID: AB_2291174
Anti-dog MHCII-APC (clone YKIX334.2)	Thermo Fisher	Cat: 17-5909-42; RRID: AB_2573242
Anti-dog CD8a-SB600 (clone YCATE55.9)	Thermo Fisher	Cat: 63-5080-42; RRID: AB_2735030
Anti-dog CD4-SB702 (clone YKIX302.9)	Thermo Fisher	Cat: 67-5040-42; RRID: AB_2744890
Anti-dog CD5-PerCP-eF710 (clone YKIX322.3)	Thermo Fisher	Cat: 46-5050-42; RRID: AB_10596668
Anti-dog CD45-eF450 (clone YKIX716.13)	Thermo Fisher	Cat: 48-5450-42; RRID: AB_10597009
Anti-human/dog CD22-PE (clone RFB-4)	Thermo Fisher	Cat: MHCD2204; RRID: AB_10372043
Anti-human/dog CD14-BV 785 (clone M5E2)	Biolegend	Cat: 301839; RRID: AB_2561366
Anti-dog/mouse FOXP3-PE-Cy7 (clone FJK-16s)	Thermo Fisher	Cat: 25-5773-82; RRID: AB_891552
Anti-dog/human Ki-67-AF 700 (clone SolA15)	Thermo Fisher	Cat: 56-5698-82; RRID: AB_2637480
Anti-human GZMB-PECF594 (clone GB11)	BD Biosciences	Cat: 562462; RRID: AB_2737618
Anti-dog Fc receptor (polyclonal)	Thermo Fisher	Cat: 14-9162-42; RRID: AB_2572935
InVivoMAb anti-mouse CD4 (clone GK1.5)	BioXcell	Cat: BE0003-1; RRID: AB_1107636
InVivoMAb anti-mouse CD8α (clone 2.43)	BioXcell	Cat: BE0061; RRID: AB_1125541
Anti-mouse IFNAR (clone MAR-1-5A3)- Purified *in vivo* Gold™ functional grade	Leinco Technologies Inc	Cat: I-401; RRID: AB_2491621
Anti-mouse IFNα (clone TIF-3C5)- Purified *in vivo* Gold™ functional grade	Leinco Technologies Inc	Cat: I-1183; RRID: AB_2737533
Anti-mouse IFNβ (clone HDβ-A47)- Purified *in vivo* Gold™ functional grade	Leinco Technologies Inc	Cat: I-1182; RRID: AB_2737532
Mouse IgG2a isotype control (clone C1.18.4)- Purified *in vivo* Gold™ functional grade	Leinco Technologies Inc	Cat: I-118; RRID: AB_2737531
InVivoMAb anti-mouse IFNγ (clone: XMG1.2)	BioXcell	Cat: BE0055; RRID: AB_1107694
InVivoMAb anti- mouse PD-1 (clone RMPI-14)	BioXcell	Cat: BE0146; RRID: AB_10949053
InVivoMAb anti-mouse CTLA-4 (clone 9D9)	BioXcell	Cat: BE0164; RRID: AB_10949609

(*Continued on next page*)

Continued		
REAGENT or RESOURCE	SOURCE	IDENTIFIER
Biological samples		
Canine PBMC	Perth Veterinary specialists	N/A
Chemicals, peptides, and recombinant proteins		
Medical grade sodium hyaluronate	Freshine Chem	Cat: FS-HA-ME0.5
High molecular weight poly(I:C)	Invivogen	Cat: tlrl-pic-5
1-ethyl-3-(-3-dimethylaminopropyl) carbodiimide hydrochloride	Sigma-Aldrich	Cat: E6383
3,3′-Dithiobis(propanoic hydrazide)	Sigma-Aldrich	Cat: 109010
Sodium phosphate	Sigma-Aldrich	Cat: 7558-79-4
Imidazole	Sigma-Aldrich	Cat: I2399
Ethylenediamine tetraacetate	Sigma-Aldrich	Cat: 03690
Cy7-NH_2	Lumiprobe	Cat: 250C0
Cy7-NHS	Lumiprobe	Cat: 25020
Cy5-NHS	Lumiprobe	Cat: 23020
Fluorescein-labelled poly(I:C)	This manuscript	N/A
Poly(I:C)-Cy5	This manuscript	N/A
Poly(I:C)-Cy7	This manuscript	N/A
Dimethyl sulfoxide (Molecular Biology)	Sigma-Aldrich	Cat: D8418
Vacmune® liquid IEX 20, GMP/clinical grade	Biosyn Corporation	N/A
Imject^{TM} mcKLH Subunits	Thermo Fisher	Cat: 77649
5,6-dimethylxanthenone-4-acetic acid (DMXAA)	Invivogen	tlrl-dmx
Recombinant mouse IFNα1	Biolegend	Cat: 751804
Recombinant mouse IFNβ1	Biolegend	Cat: 581304
Dulbecco's PBS	Merck	Cat: MS-012-A
RNAlater^{TM} Stabilization Solution	Invitrogen	Cat: AM7021
TRIzol^{TM} Reagent	Invitrogen	Cat: 15596026
Histopaque	Sigma-Aldrich	Cat: 10771
Critical commercial assays		
Foxp3/Transcription Factor Staining Buffer Set	eBioscience	Cat: 00-5523-00
RNEasy Mini Kit	Qiagen	Cat: 74104
Tumor dissociation Kit, mouse	Miltenyi Biotec	Cat: 130-096-730
CRISPRMAX Cas9 Transfection Reagent	Thermo Fisher	Cat: CMAX00008
Milliplex canine Cytokine magnetic bead panel	Merk Millipore	Cat: CCYTOMAG-90K
CFSE labeling kit	Thermo Fisher	Cat: C34554
Deposited data		
Raw and analyzed bulk RNAseq data (mouse)	This manuscript	GEO: GSE229021, GSE229950, GSE230269
Experimental models: Cell lines		
WEHI 164	CellBank Australia	N/A
AE17	CellBank Australia	N/A
M3-9-M	CellBank Australia	N/A
CT26	NIH Division of Cancer Treatment and Diagnosis	N/A
WEHI 164 TLR3 K/O	This manuscript	N/A
Experimental models: Organisms/strains		
Mouse: BALB/cArc	Animal Resource Center (Murdoch, WA, Australia)	Product code: BC
Mouse: BALB/cJAusb	Australian BioResources (Moss Vale, NSW, Australia)	N/A
Mouse: BALB/cJAusb	Harry Perkins Institute Medical Research (Nedlands, WA, Australia)	N/A

(*Continued on next page*)

Continued

REAGENT or RESOURCE	SOURCE	IDENTIFIER
Mouse: C57BL/6J	Animal Resource Center (Murdoch, WA, Australia)	Product code: B6<br>JAX stock number: 000,664
Oligonucleotides		
Guide targeting exon 4 of TLR3 gene<br>5′ CGTTGTATCTCACAGTGCAT 3′	Integrated DNA Technologies	N/A
Alt-R® CRISPR-Cas9 tracrRNA	Integrated DNA Technologies	Cat: 1072534
Alt-R® CRISPR-Cas9 negative control crRNA	Integrated DNA Technologies	N/A
Fwd_OT1-2 TLR3<br>5′ CCGGTGCAGTAACCAACCTA 3′	Integrated DNA Technologies	N/A
Rvse_OT1-2 TLR3<br>‘5 TGGTGGATGCAAACCCCCAG 3’	Integrated DNA Technologies	N/A
Fwd_OT3 TLR3<br>5′ CTTTGGGTCTCCACACAACAA 3′	Integrated DNA Technologies	N/A
Rvse_OT3 TLR3<br>5′ GGTCTGGCACCTATGAGTTTT 3′	Integrated DNA Technologies	N/A
Fwd_OT4 TLR3<br>5′ TTGCTTTTCACGAGCCAGTG 3′	Integrated DNA Technologies	N/A
Rvse_OT4 TLR3<br>5′ GTGGGGAAGAGCGAGCAAG 3′	Integrated DNA Technologies	N/A
Fwd_OT5 TLR3<br>5′ AAGAAGTGGTGGGTGCTCTG 3′	Integrated DNA Technologies	N/A
Rvse_OT5 TLR3<br>5′ TGTAGAATTTCTGAATTGCTCTATGAT 3′	Integrated DNA Technologies	N/A
Fwd_OT6 TLR3<br>5′ TGAGAGTGTGTTTGCTGGCT 3′	Integrated DNA Technologies	N/A
Rvse_OT6 TLR3<br>5′ CAGGTGCTAGTTCAGGTCCA 3′	Integrated DNA Technologies	N/A
Software and algorithms		
FlowJo v10	Becton, Dickinson	https://www.flowjo.com
GraphPad Prism v9	GraphPad Software	https://www.graphpad.com
FastQC v0.11.3	Andrews[58]	https://www.bioinformatics.babraham.ac.uk/projects/fastqc/
Kalisto v0.43.0	Bray et al.[59]	https://doi.org/10.1038/nbt.3519
DESeq2	Love et al.[60]	https://doi.org/10.1186/s13059-014-0550-8
GSEA	Subramanian et al.[61]	https://doi.org/10.1073/pnas.0506580102
Imsig	Nimral et al.[35]	https://doi.org/10.1158/2326-6066.CIR-18-0342
CIBERSORTx	Newman et al.[38]	https://doi.org/10.1038/nmeth.3337
ggplot2 v3.3.6	Wickham[62]	N/A
Other		
Roswell Park Memorial Institute (RPMI) 1640	Invitrogen	Cat: 11875119
Fetal bovine serum	CellSera	Cat: AU-FBS/PG
HEPES (1 M)	Thermo Fisher Scientific	Cat: 15630080
2-Mercaptoethanol (1000x)	Thermo Fisher	Cat: 2198023
Penicillin-Streptomycin (10,000 U/ml)	Thermo Fisher Scientific	Cat: 15140122
Trypsin-EDTA (0.5%)	Thermo Fisher Scientific	Cat: 15400054
Dimethyl sulfoxide (Cell culture)	Sigma-Aldrich	Cat: D2650
Dulbecco’s PBS	Merck	Cat: MS-012-A
RNAlater™ Stabilization Solution	Invitrogen	Cat: AM7021
TRIzol™ Reagent	Invitrogen	Cat: 15596026
Histopaque	Sigma-Aldrich	Cat: 10771

RESOURCE AVAILABILITY

Lead contact

Further information and requests for resources and reagents should be directed to and will be fulfilled by the lead contact, Willem Joost Lesterhuis willem.lesterhuis@uwa.edu.au.

Materials availability

The TLR3 K/O WEHI 164 cell line generated in this study can be obtained from our lab.

Data and code availability

- Bulk RNA sequencing data have been deposited at GEO and are publicly available as of the date of publication via accession numbers: GEO: GSE229021, GSE229950, GSE230269.
- This paper does not report original code.
- Any additional information required to reanalyze the data reported in this paper is available from the lead contact upon request.

EXPERIMENTAL MODEL AND SUBJECT DETAILS

Mice

All mouse studies were approved by the ethics committee of the Harry Perkins Institute of Medical Research (Animal ethics protocol numbers: AE123, AE124, AE161, AE191, AE220). Male and female BALB/cArc, BALB/cJAusb, and C57BL/6J mice aged 8–12 weeks were purchased from the Animal Resource Center (Murdoch, WA), or Australian BioResources (Moss Vale, NSW), or the Harry Perkins Institute of Medical research (Nedlands, WA) and maintained under pathogen-free conditions at the Bioresources facility at the Harry Perkins Institute of Medical Research. Mice were fed Rat and Mouse cubes (Specialty Feeds, Glen Forrest, Australia) and had access to water *ad libitum*. Cages (Techniplast, Italy) were individually ventilated with filtered air, contained aspen chips bedding and were supplemented with tissues, cardboard rolls and wood blocks as environmental enrichment, and were changed every 14 days. Mice were housed at 21–22°C, 60% humidity with 12 h light/dark cycle (06:00–18:00).

All experiments were carried out in accordance with the Australian Code for the Care and Use of Animals for Scientific Purposes (eighth Edition, 2013) and following the institutional guidelines from the Animal Ethics Committee at the Harry Perkins Institute of Medical Research and conformed to the National Health and Medical Research Council guidelines for the care of use of laboratory animals.

Canine subjects

Canine veterinary patients with an existing soft tissue sarcoma or mast cell tumor that required surgical resection were recruited to the study at Perth Veterinary Specialists, Osborne Park, Perth, Western Australia. Ethics approval was obtained via the Murdoch University (Murdoch, WA) Animal Ethics Committee (Protocol ID: 871, Permit No. R3340/21). We included three dogs with the following patient characteristics: an 8-year-old Labrador of 30.7kg with a mast cell tumor on its chest; a 12-year-old Staffordshire Terrier of 16.9 kg with a grade 1 soft tissue sarcoma on its forelimb; and a 9-year-old Shih Tzu of 11 kg with a grade 1 soft tissue sarcoma on its distal limb.

Cell lines

Cell lines WEHI 164, M3-9-M, and AE17 were obtained from CellBank Australia (Westmead, NSW). Cell line CT26 was obtained from the NIH Division of Cancer Treatment and Diagnosis tumor repository. The TLR3 K/O WEHI 164 cell line was prepared by deleting the TLR3 gene from the wild type WEHI 164 using CRISPR/Cas9 system (Integrated DNA Technologies) with a TLR3 specific guide RNA. All cell lines were maintained in Roswell Park Memorial Institute (RPMI) 1640 (Invitrogen) supplemented with 10% FBS (Fisher Biotech), 20mM HEPES (Invitrogen), 0.05 mM 2-mercaptoethanol, and 100 U/ml penicillin (Invitrogen). Cells were passaged for three to five times before animal inoculation and were tested for mycoplasma for every six months and remained negative. Cell lines were validated for MHC-I molecules H2-kb (C57BL/6J) or H2-kd (BALB/c) yearly.

METHOD DETAILS

Preparation of hydrogels

Medical grade sodium hyaluronate of intrinsic viscosity 0.5–1.0 m^3/kg and molecular weight 20–60 kDa (FS-HA-ME0.5) was obtained from Freshine Chem/Bloomage Biotech (Jinan, China). High molecular weight poly(I:C) was obtained from Invivogen (tlrl-pic-5, 1.5–8 kbp). Other chemicals were obtained from Merck (Sigma-Aldrich) Australia unless specified.

The hyaluronic acid (HA) was modified with 3,3′-Dithiobis(propanoic hydrazide) (DTPH) to form thiol-modified HA as previously described.[19] Briefly, in a typical preparation of thiol-modified HA, sodium hyaluronate (1 g, 2.49 mmol repeating unit) was treated with DTPH crosslinker (22.4 mg, 0.036 eq) in the presence of 1-ethyl-3-(-3-dimethylaminopropyl) carbodiimide hydrochloride

(EDC) (16.8 mg, 0.035 eq) at pH 4.75 at 25°C overnight. HCl solution (0.1 M) was added as necessary to maintain the pH. The pH was raised to 7.0 with 0.1 M NaOH before the addition of dithiothreitol (DTT; 87.5 mg, ~16 eq relative to EDC). The pH was raised to 8.5 with 0.1 M NaOH and left for 3.5 h to reduce disulfides to free thiols. The product was dialyzed against 0.1 M NaCl adjusted to pH 3.75 with 1 M HCl and then deionized water, before being lyophilized. HA-DTPH was stored in a desiccator at room temperature. HA with different mol % crosslinker were prepared similarly, scaling the amount of DTPH and EDC proportionally.

To form HA hydrogel, in a representative preparation of a 2.5% w/v hydrogel, 950 μL water and 50 μL 10x PBS were added to 25 mg HA-DTPH in this order and mixed by gentle inversion for 1–2 h until completely dissolved, yielding a colorless, clear, viscous solution. Then, 50 μL dimethyl sulfoxide (DMSO) were added, and the mixture was thoroughly combined by inversion until homogeneous followed by a brief centrifugation to collect the contents and remove any bubbles. Poly(I:C)-loaded hydrogels for *in vivo* anti-tumor efficacy experiments were prepared as above. Hydrogels were aliquoted and/or cast as required and allowed to set for 48 h at room temperature, before being stored at 4°C. Hydrogel discs for mechanical studies were cast in 24-well plates in triplicate (~3 mL per well), covered with parafilm, and allowed to set before being carefully removed from wells and maintained in a humidified environment until ready for use. For *in vitro* and *in vivo* gel degradation studies, HA polymers were modified using Cy7-NH2 (Lumiprobe, cat: 250C0) in place of DTPH and stored protected from light in a desiccator at room temperature. Hydrogels were prepared in the same way as described above, substituting 10% by weight of the HA-DTPH with HA-Cy7. For *in vitro* and *in vivo* release studies, poly(I:C) (Invivogen, cat: tlrl-pic-5) was fluorescently tagged with Cy7 or Cy5 using Cy7-NHS (Lumiprobe, cat: 25020) or Cy5-NHS (Lumiprobe, cat: 23020), respectively, before being encapsulated in HA hydrogels. To fluorescently tag poly (I:C), poly(I:C) was dissolved in water at 5 mg/mL and then 0.25 volumes of 5x reaction buffer (100 mM sodium phosphate pH 7.2, 100 mM EDTA, 1.5 M NaCl) was added followed by 3.5 mg of 1-ethyl-3-(3-dimethylaminopropyl) carbodiimide (EDC). Then, 50 μL of a solution containing 100 mM imidazole and 250 mM ethylenediamine was added. A further 50 μL of 100 mM imidazole was added, and the mixture was left overnight at 25°C. The product was dialyzed against 1x reaction buffer before 100 μL of Cy7-NHS or Cy5-NHS (1 mg/mL in DMSO) were added and left at 25°C overnight. The product was then dialyzed thoroughly against water to remove unreacted Cy7-NHS or Cy5-NHS, before addition of 0.1 volumes 5 M NaCl followed by 2.75 volumes ethanol. The conjugate was centrifuged at 20,000g for 10 min, washed with 70% ethanol, and redissolved in water at 55°C. The concentration of the conjugate was measured by spectrophotometry (Nanodrop) using a nucleic acid extinction coefficient of 90 ng-1 μL cm-1 at 260 nm.

Characterization of mechanical properties of hydrogel

Characteristic stress-strain curves of different hydrogels were produced by uniaxial compression testing using a custom-built compressive system.[63] Cylindrical, 15 mm-tall, 15 mm-diameter, hydrogel discs were compressed against a flat plate by a mechanical translation stage at a rate of 0.5% axial strain per second, up to 50%, at room temperature. The resulting force from this deformation was measured by a load cell (LSB200, FUTEK Advanced Sensor Technology, Inc.) which, along with knowledge of the geometric cross-section of the sample, allowed stress to be determined. To achieve enough precision, measurements were performed on three independent samples three times for each of the three replicates. Measurements were fitted to a double-exponential curve and Young's modulus was measured by dividing the axial stress by strain, at each point along the stress-strain curve.

Hydrogel mechanical properties at a microscale level were characterized by quantitative micro-elastography (QME).[20] QME is a variant of compression optical coherence elastography (OCT) that maps tangent modulus throughout a sample volume on the microscale level.[20] In QME, the sample is compressed and the resulting sample deformation is measured using OCT. This deformation is related to Young's modulus using a mechanical model. Young's modulus is mapped at each voxel in the OCT field of view. QME measurements were performed using a fiber-based spectral-domain OCT system (Thorlabs Inc., USA). In this custom-built QME system, the light source is a superluminescent diode with a mean wavelength of 1300 nm and a spectral bandwidth of 170 nm (full-width at half maximum (FWHM)). The measured OCT axial and lateral resolutions (FWHM) in air were 4.8 μm and 7.2 μm, respectively. In a typical experiment, cylindrical 3 mm-tall, 10 mm-diameter HA gel discs were prepared for improved compatibility with the QME system and were placed between a rigid glass imaging window (Edmund Optics Inc., USA) and a motorized translation stage (Thorlabs Inc., USA). The imaging window, through which the OCT beam illuminates the sample, was fixed to an annular piezoelectric actuator. A 250 μm thick compliant silicone layer (Wacker, Germany) was placed between the HA gel disc and imaging window. Silicone oil was used to lubricate both the hydrogel and compliant layer to reduce friction. Using the translation stage, a preload strain of 5–10% was applied to the HA gel to ensure uniform contact. A micro-scale compression was applied using the piezoelectric actuator (Piezomechanik GmbH, Germany). Axial displacement in the compliant layer and HA gel at each voxel in the OCT field of view was measured from changes in the phase of the OCT signal. The piezoelectric actuator was driven by a 12.5 Hz square wave, collinearly with the OCT beam, and synchronized with the acquisition of OCT B-scans. Two B-scans were acquired for each lateral *y*-location such that alternate B-scans were acquired at different micro-scale compression levels. OCT scans comprised 1000 A-scans per B-scan and 1000 B-scan pairs per volume over a 3 mm × 3 mm (*x*,*y*) field of view in the center of each HA gel disc. Laterally scanning the beam enables three-dimensional (3-D) images of the sample microstructure to be generated to approximately 2.5 mm in depth. Assuming an HA gel refractive index of 1.4, the resulting (*x*,*y*,*z*) voxel size was 3 μm × 3 μm x 2.5 μm. Local axial strain was calculated from the gradient of axial displacement with depth using one-dimensional weighted least squares linear regression over a sliding window of 100 μm. The preload strain at each lateral location in the compliant layer was measured using OCT as the change in layer thickness divided by the initial layer thickness. The stress-strain curve of the compliant layer was measured using uniaxial compression testing apparatus described in STAR Methods section of this manuscript. Local axial stress in the compliant layer from the

CellPress
OPEN ACCESS

micro-scale actuation was calculated by multiplying local axial strain in the compliant layer by the tangent modulus (*i.e.*, the gradient of the tangent of the stress-strain curve) at the preload strain. Assuming uniaxial stress, tangent modulus in the sample was calculated by dividing the local axial strain in the HA gel by the axial stress at the HA gel surface. At low preload strains, tangent modulus is equivalent to Young's modulus.

In vitro release study

To determine the release kinetics of poly(I:C) from hydrogel, HA hydrogel loaded with poly(I:C)-Cy5 were aliquoted (50 μL per well in a clear 96-well plate) and allowed to set for 48 h at 25°C. 200 μL release media (PBS +100 U/ml penicillin +100 μg/mL streptomycin) supplemented with hyaluronidase were added to each well and incubated at 37°C protected from light. After the desired release time had elapsed, a 105 μL aliquot of release media was collected from each well and stored at −20°C until quantified. To quantify, 100 μL aliquots of release media were placed in a 96-well black well plate and measured by plate reader (610/675 nm). Data were normalized relative to a solution of 50 ng/μL Cy5-labeled poly(I:C) (corresponding to 100% release). All gels and release conditions were measured in triplicate.

In vivo degradation study and drug-release profile

To test the degradation speed of the hydrogel, Cy7-labeled hydrogel was implanted in the subcutaneous wound cavity following a 1 cm skin incision on the right flank of mice to create a lateral subcutaneous pocket. To evaluate the *in vivo* release of poly(I:C), the hydrogel containing Cy7-labeled poly(I:C) was implanted in the wound cavity using the skin incision model as described above. Cy7-labeled hydrogels containing unlabeled poly(I:C) were used as controls to assess the effect of poly(I:C)-recruited immune cells on gel degradation speed. Fluorescence signal was monitored by imaging mice using the CRI Maestro2 *in vivo* imaging system using the near infrared (NIR) emission filter. The fluorescent signal was quantified using the CRi Maestro Software. Composite cubes were unmixed using Compute Spectra tool and the fluorescence signal was expressed as total signal (x10^6phot/cm^2/s). The percentage remaining signal relative to the signal at day 0 was plotted over time.

Surgical utility of hydrogels

To assess the physical consistency of the hydrogels, we performed visual inspection of the hydrogels following expulsion from a syringe and by applying the hydrogels in a Petri dish. To assess the surgical utility, we applied the hydrogels in mice in a subcutaneous resection site and assessed adherence to tissue in the wound area. We visually assessed hydrogel leakage or lack thereof upon wound closure as per our surgical protocol.

Intratumoral therapy for subcutaneous model

Once cell lines were 70–80% confluent, they were harvested and washed three times in sterile 1x phosphate buffer saline (PBS). A total of 5x10^5 cells in 100 μL PBS were inoculated subcutaneously into the shaved lower right flank of mice using a 26G needle. Mice were randomized when tumors became palpable (day 3–5) and once tumors were established, mice were treated intratumorally with immunotherapy in a 25 μL injection, daily for 3 days (q1dx3) or 6 days (q1dx6) or 14 days (q1dx14). The following dosages of immunotherapies were used: Poly(I:C) at 1 μg/day, low dose; or 10 μg/day, medium dose; or 50 μg/day, high dose), DMXAA at 50 μg/day, rIFNα at 2000 IU/day, and rIFNβ at 2000 IU/day. All immunotherapies were resuspended in sterile endotoxin-free Dulbecco's PBS (1x) (Merck). Tumors were measured using a caliper three times weekly and tumor sizes were determined by computing the product of length and width. Mice were euthanized once tumors reached a size of 100 mm^2.

In vivo wound healing model

A full-thickness skin incision was performed on the right flank of mice under sterile conditions. Mice were dosed with buprenorphine (0.1 mg\kg) in 100 μL subcutaneously 30 min before surgery. Mice were then anesthetized using isoflurane (4% in 100% oxygen at a flow rate of 1 L/min induction, and 3–3.5% isoflurane in 100% oxygen at a flow rate of 0.5 L/min maintenance). The incision site was shaved and cleaned with chlorhexidine 100% followed by 0.5% chlorhexidine in 70% ethanol. A 1 cm full-thickness incision was made using scissors. A ruler was used to standardize the size of the incision across all mice to 1 cm. The wound was closed with a tissue glue (3M, Vetbond). Starting from the day of surgery, mice were dosed with either poly(I:C), 10 μg/day; DMXAA, 50 μg/day; rIFNα, 2000 IU/day; or rIFNβ 2000, IU/day in a final volume of 50 μL endotoxin free DPBS in the wound area, daily, for 4 days (q1dx4). Photographs were taken daily and on day 4 (the timepoint at which the difference between treatment groups were clear) mice were euthanized, and wound skin samples along with surrounding healthy skin were harvested. Wound skin samples were fixed in 4% formaldehyde, processed using the Leica tissue processor and then embedded in paraffin. Paraffin-embedded tissue blocks were sectioned into 4 μm skin sections and stained with hematoxylin and eosin. Stained slides were scanned using the 3DHISTECH slide scanner.

Anti-tumor efficacy of hydrogel-poly(I:C) after incomplete tumor resection

The mouse model of incomplete tumor resection was used as previously described.[30] Briefly, once tumors were established (25–35 mm^2 for WEHI 164, 16–25 mm^2 for CT26 or M3-9-M), mice were dosed subcutaneously with buprenorphine (0.1 mg/kg in 100 μL) 30 min before surgery. Mice were anesthetized using isoflurane (4% in 100% oxygen at a flow rate of 1 L/min induction and 4% isoflurane in 100% oxygen at a flow rate of 0.5 L/min maintenance). 75% or 90% of the tumor bulk was removed and

100 μL of empty hydrogel or poly(I:C)-loaded hydrogel was applied in the wound bed before it was closed using surgical clips (Reflex 7mm Clips, cat# AS59038, Able scientific, Australia) or tissue glue (3M, Vetbond). In the C57BL/6 tumor model (M3-9-M), mice were additionally bandaged in order to prevent them opening their wounds. Surgically removed tumors were immediately placed in RNAlater (Invitrogen) for RNAseq studies. After surgery, mice were immediately placed in a heat box at 37°C and monitored for recovery. Mice were dosed subcutaneously with buprenorphine (0.1 mg/kg in 100 μL) at the end of the day and 24 h thereafter. Mice were monitored for tumor recurrence. Recurred tumors were measured three times weekly using a caliper and tumor sizes were determined by computing the product of length and width. Mice were euthanized once tumors reached a size of 100 mm^2. Depending on the experimental question and the model used, we performed different levels of tumor debulking. The CT26 colorectal cancer and M3-9-M rhabdomyosarcoma models grow more rapidly and are less immunogenic than the WEHI 164 fibrosarcoma model. Therefore, we removed less tumor bulk in WEHI 164 (75% debulk) than in CT26 or M3-9-M (90% debulk) when studying the efficacy of poly(I:C) hydrogel. In experiments testing the efficacy of immune checkpoint therapy in combination with the poly(I:C) hydrogel, we removed less tumor bulk (75% debulk) in order to achieve a low background response for either treatment alone, allowing proper assessment of the interaction of the two treatments.

In vivo ICT treatment

Following incomplete tumor resection, mice received an intraperitoneal (i.p.) dose of 200 μg anti-PD-1 (BioXcell, Clone RMPI-14) starting on day 3 of surgery and then two additional doses at day 5 and 7 relative to surgery. A separate group of mice received a single intraperitoneal dose of 100 μg anti-CTLA-4 (BioXcell, Clone 9D9) starting on day 3 of surgery. In previously published studies by our group,[64] we did not observe any difference between using isotype controls versus PBS; therefore, control mice received PBS alone in these experiments.

In vivo antibody blocking

Mice with established WEHI 164 tumors were treated with cytokine neutralizing monoclonal antibodies starting one day prior to intratumoral poly(I:C) (10 μg/mouse/day, q1dx6) and then every 3 days for a total of 3 doses. Antibodies were delivered in 100 μL of PBS, i.p., at the following dosages: for anti-IFNAR (clone MAR-1-5A3), 0.5 mg/mouse/day; anti-IFNα (clone TIF-3C5), 1 mg/mouse/day; anti-IFNβ (clone HDβ-4A7), 0.6 mg/mouse/day; mouse IgG2a isotype control (clone C1.18.4), 0.5 mg/mouse/day; all from Leinco Technologies Inc., and anti-IFNγ (clone XMG1.2), 0.5 mg/mouse/day, from BioXcell.

In vivo cell depletion

Mice with established WEHI 164 tumors were treated with cell depleting monoclonal antibodies starting one day prior to treatment with intratumoral poly(I:C) (10 μg/mouse/day, q1dx6). Anti-CD4 (clone GK1.5) and anti-CD8α (clone 2.43) were each administered at 100μg/mouse in 100 μL of PBS, i.p., every 3 days for a total of 3 doses.

In vivo cellular uptake of poly(I:C)

Mice with established (25–35 mm^2) WEHI 164 tumor were dosed intratumorally with 50 μg of fluorescein-labeled poly(I:C) in 25 μL of PBS injection. In a separate group of mice, we investigated poly(I:C) uptake over time (time point day 1, early timepoint; and day 5, late timepoint), after daily injections of unlabeled poly(I:C) followed by one injection of fluorescein-labeled poly(I:C). Tumors were harvested 1 h post injection of fluorescein-labelled poly(I:C) and processed for flow cytometry.

Flow cytometry staining and FACS analysis

For flow cytometry, tumors were harvested and immediately submerged in cold PBS and kept on ice. Tumors were cut into small pieces on a Petri dish using a scalpel blade and digested using the gentle MACS system (Miltenyi Biotec). Samples were washed three times with 1x PBS before adding FC block (anti-CD16/CD32, Becton Dickinson) (dilution 1:1000) for 20 min on ice. Fixable Viability Stain 780 (dilution 1:1000) was used to discriminate live from dead cells. Antibodies for surface staining were resuspended in FACS buffer (cold PBS with 2% v/v FBS, and 2 mM EDTA) and incubated for 30 min at RT. For intracellular FoxP3 staining, cells were permeabilized and fixed using the FoxP3 transcription factor Fix/Perm buffer kit (eBioscience) following manufacturer guidelines. Cells were washed three times and resuspended in stabilizing fixative until ready for acquisition. Before acquisition, samples were resuspended in FACS buffer (cold PBS with 2% v/v FBS, and 2 mM EDTA). Data were acquired on BD FACS LSR Fortessa and analyzed using FlowJo (V10.8.1). See Figure S6 for gating strategies. For cytometry antibodies are detailed in the key resources table.

RNA-seq extraction

For assessment of the effect of poly(I:C), WEHI 164 tumors were treated intratumorally with 1 μg poly(I:C), daily, for up to 6 days (q1dx6). Tumors were harvested at pre-determined timepoints: Day 0 (untreated), 1, 3, 5 or 7 days after the first dose of poly(I:C). The surrounding tissue was removed, and tumors immediately submerged in RNAlater (Invitrogen). To identify pre-treatment factors which determine the efficacy of poly(I:C) hydrogel after incomplete tumor resection, resected tumors (75–90%) were placed immediately into RNAlater (Invitrogen). All samples were stored at 4°C for 24 h, after which supernatant was removed and samples transferred to −80°C. Frozen tumors were dissociated in Trizol (Invitrogen) using a TissueRuptor (QIAGEN). RNA was extracted using chloroform and purified on RNeasy MinElute columns (QIAGEN). RNA quantity and quality was measured and confirmed using nanodrop

(Thermofisher). Library preparation and sequencing (100 bp, single end, for poly(I:C) for time course WEHI 164 tumors; and 150 bp, paired end, for surgically resected WEHI 164 and CT26 tumors) was performed by Australian Genome Research Facility, using Illumina HiSeq standard protocols.

Deletion of TLR3 by CRISPR/Cas9

To create a genomic knockout of TLR3 in WEHI 164 cell line, we used the Alt-R CRISPR-Cas9 System (Integrated DNA Technologies) as previously described.[65] Briefly, a ribonucleoprotein (RNP) complex was formed through the combination of the predesigned crRNA (Ordered through IDT; www.idtdna.com/CRISPR-Cas9), complexed with a tracrRNA and the Cas9 nuclease. The RNP complex was then delivered into cancer cells through lipofection using Lipofectamine CRISPRMAX Cas9 Transfection Reagent (Thermo Fisher). The crRNA, to protospacer 5′ CGTTGTATCTCACAGTGCAT 3′, targeting the TLR3 gene exon 4 was used to guide the Cas9 and to induce DNA double-strand breaks, resulting in frameshift truncation genomic editing. An amplicon sequencing method was used to validate genomic editing as previously described.[65]

The following top six predicted off-target sites were sanger sequenced to confirm integrity: target sequence 5′ GGTGGTATCTCCCAGTGCAT 3′, targeting both *APOL9A* (locus: chr15:-77406483) and *APOL9B* (chr15:+77733679) genes was amplified using the Fwd_OT1-2 TLR3 (5′ CCGGTGCAGTAACCAACCTA 3′) and Rvse_OT1-2 TLR3 (‘5 TGGTGGATGCAAAC CCCAG 3’) primers; target sequence 5′ GGTTCTCTCTCACAGTGCAT 3’ (locus: chr9:-8825905) was amplified using the Fwd_OT3 TLR3 (5′ CTTTGGGTCTCCACACAACAA 3′) and Rvse_OT3 TLR3 (5′ GGTCTGGCACCTATGAGTTTT 3′) primers; target sequence 5′ AGCTGTAT-TCACAGTGCAT 3’ (chr9:-123159523) was amplified using the Fwd_OT4 TLR3 (5′ TTGCTTTTCACGAGCCAGTG 3′) and Rvse_OT4 TLR3 (5′ GTGGGGAAGAGCGAGCAAG 3′) primers; target sequence 5′ CACTGTATCT-ACAGTGCAT 3’ (chr16:-40725300) was amplified using the Fwd_OT5 TLR3 (5′ AAGAAGTGGTGGGTGCTCTG 3′) and Rvse_OT5 TLR3 (5′ TGTAGAAT TTCTGAATTGCTCTATGAT 3′) primers; and finaly target sequence: 5′ CCCTGTATCTAACAGTGCTT 3’ (locus: chrX:+89908686) amplified using Fwd_OT6 TLR3 (5′ TGAGAGTGTGTTTGCTGGCT 3′) and Rvse_OT6 TLR3 (5′ CAGGTGCTAGTTCAGGTCCA 3′) primers.

Surgical feasibility in canine soft tissue cancer

To assess the surgical utility and usability of the poly(I:C) hydrogel in an oncological surgical setting, we established a feasibility study in canine soft tissue cancer. Canine veterinary patients with an existing soft tissue sarcoma or mast cell tumor that required surgical resection were recruited to this study at Perth Veterinary Specialists, Osborne Park, Perth, Western Australia. Patients received intra-operative poly(I:C)-hydrogel, containing 0.2 mg poly(I:C) and 1 mg KLH subunits (Vacmune liquid IEX 20, GMP/clinical grade, Biosyn), at the time of surgery. Patient blood samples were taken directly prior to surgery, and at 2 weeks and 3 months post-surgery, and analyzed at VetPath (Perth, Western Australia) using the standardized CP2 complete canine profile to measure blood parameters.

Canine PBMC isolation

Canine blood samples were collected in heparinized vacutainer tube (Becton Dickinson) and couriered to the Telethon Kids Institute (Perth, Western Australia) for PBMC isolation. Briefly, whole blood was diluted 1:2 in sterile PBS and layered onto Histopaque (Sigma Aldrich) for density gradient separation of PBMCs using the following centrifugation settings: 400 x g for 30 min, at RT, 9-accelerate, 0-decelerate-no break. Isolated PBMCs were washed twice in sterile RPMI1640 media (Invitrogen) (centrifugation settings: 400 x g for 10 min, at RT, 9-accelerate, 1-decelerate-no break) before counting and cryopreservation in freezing media (RPMI 164 media containing 20% FCS and 10% DMSO).

KLH T cell proliferation assay

Canine PBMCs were thawed and rested for 1 h at 37°C prior to CFSE labeling by incubating with 10mM CFSE (Thermo Fisher) for 10 min at 37°C while protected from light. Cells were washed twice in RPMI1640 media and counted. $2x10^5$ CFSE-labeled PBMCs were plated per well in 200 μL of complete culture media: RPMI 1640 (Invitrogen) supplemented with 10% FBS (Fisher biotech), 20mM HEPES (Invitrogen), 0.05 mM 2-mercaptoethanol (Thermo Fisher), and 100 U/ml penicillin (Invitrogen) with or without KLH protein (20 μg/mL, Imject mcKLH Subunits, Thermo Fisher). 72 h later, cells were analyzed by flow cytometry for CFSE dilution and intracellular cytokine staining using a panel of canine antibodies derived from OMIP-065.[66] Flow cytometry antibodies are detailed in the key resources table.

Canine luminex multiplex cytokine assay

Culture supernatants were collected from T cell proliferation assays at 48 h timepoints, spun down to remove debris and stored at −80°C. 25uL of culture supernatants were run in duplicate, alongside assay standards and QC samples, using the Milliplex Canine Cytokine/Chemokine Magnetic Bead Panel (Merck) on the Bio-Ple×200 system (Bio-Rad) following the manufacturers guidelines. Samples were assayed at 1:1 and 1:10 dilution in assay matrix to ensure they fell within the limit of detection of the assay. Sample data readings were fitted to the standard curves for quantification using the Bioplex Manager software (v6.2, Bio-Rad).

QUANTIFICATION AND STATISTICAL ANALYSIS

Analysis of RNAseq data

For the time-course poly(I:C) samples, a total of 15 WEHI 164 tumor samples across five time points were processed. For the surgically resected tumors, mice were monitored after application of the poly(I:C) hydrogel to determine if they were a responder (complete regression) or non-responder (no delay in growth). From the CT26 (90% debulk), a total of 6 responders and 4 non-responders were selected. From the WEHI 164 (75% debulk), a total of 5 responders and 5 non-responders were selected. After reviewing quality control on all samples using FastQC software (v0.11.3),[58] Kallisto (v0.43.0)[59] was used for transcript abundance estimation. Transcript-to-gene mapping was performed using tximport using the mm10 reference. To obtain the dynamic gene expression data, we first filtered data to retain genes with a count per million (CPM) greater than 0.366 in at least 3 samples. Samples were normalized using the variance stabilizing transformation (VST) method. The top 5000 genes were selected, centered, and normalized around the median and plotted in a heatmap using ggplot2. We clustered time course RNAseq data using the fuzzy c-means (FCM) clustering algorithm Mfuzz[67] in the TCseq package.[68] TCseq is a computational RNAseq analysis tool that allows interrogation of time course gene expression data. Specifically, it clusters genes that have identical behavior in time. A TCseq plot shows the *Z* score of each gene within a cluster as a line across multiple time points. Once those clusters have been identified, the biological relevance of those clusters can be further interrogated by conventional pathway analysis tools such as Gene Ontology (GO). Z-normalized/scaled counts were used in the algorithm and expression profiles were grouped in clusters ($k = 6$) based on their dynamic patterns. Genes belonging to each cluster were extracted and enrichment of per-cluster genes was performed using Gene Ontology.

We used a deconvolution approach to deduce the cell subtypes present at each time point. The Imsig algorithm[35] or CIBERSORTx algorithm[38] was used to estimate the relative proportions of immune cell types based on the transcriptomic profiles of each sample, using the inbuilt reference. Before analysis, transcript-level data were library-sized, and gene length normalized to TPM.

We used the Broad Institute GSEA software[61] to analyze normalized gene expression data to compare responders and non-responders (collapse = false). We used the hallmarks gene set database, which includes 50 MSig DB hallmarks gene sets[69] to analyze upregulated pathways in responders versus non responders. We set a threshold of a nominal $p < 0.005$ and FDR< 0.25 for significant gene set enrichments.

Statistics and data analysis

R (v4.1.0) was used for analysis of RNAseq data. Graphs were plotted using the ggplot2 package (v3.3.6).[62] For tumor growth curves and survival, GraphPad Prism (v9) was used for plotting data and statistical analysis. Survival analyzes were performed using Kaplan Meier method and test for significance were determined by non-parametric, two-tailed log rank (Mantel-Cox) test. Unpaired Student's t tests were used for two group analyzes. For statistical significance, the p value of compared groups were at $^{*}p \leq 0.05$, $^{**}p \leq 0.01$, $^{***}p \leq 0.001$, $^{****}p \leq 0.0001$. FACS data were analyzed using FlowJo (v10).

Mice were randomized to different treatment groups before the start of treatment. For experiments assessing treatment efficacy, a sample size of n = 8–10 animals was required to detect an increase in response from 0% for incomplete surgical resection alone to 60% for experimental arms with a power of 0.8% and an alpha of 0.05, using a chi-square test.

Cell Reports Medicine, Volume *4*

Supplemental information

A surgically optimized intraoperative poly(I:C)-releasing hydrogel prevents cancer recurrence

Francois Xavier Rwandamuriye, Cameron W. Evans, Ben Wylie, Marck Norret, Breana Vitali, Diwei Ho, Dat Nguyen, Ellise A. Roper, Tao Wang, Matt S. Hepburn, Rowan W. Sanderson, Maren Pfirrmann, Vanessa S. Fear, Catherine A. Forbes, Ken Wyatt, Anne L. Ryan, Terrance G. Johns, Marianne B. Phillips, Rupert Hodder, Connull Leslie, Brendan F. Kennedy, Rachael M. Zemek, Killugudi Swaminathan Iyer, and Willem Joost Lesterhuis

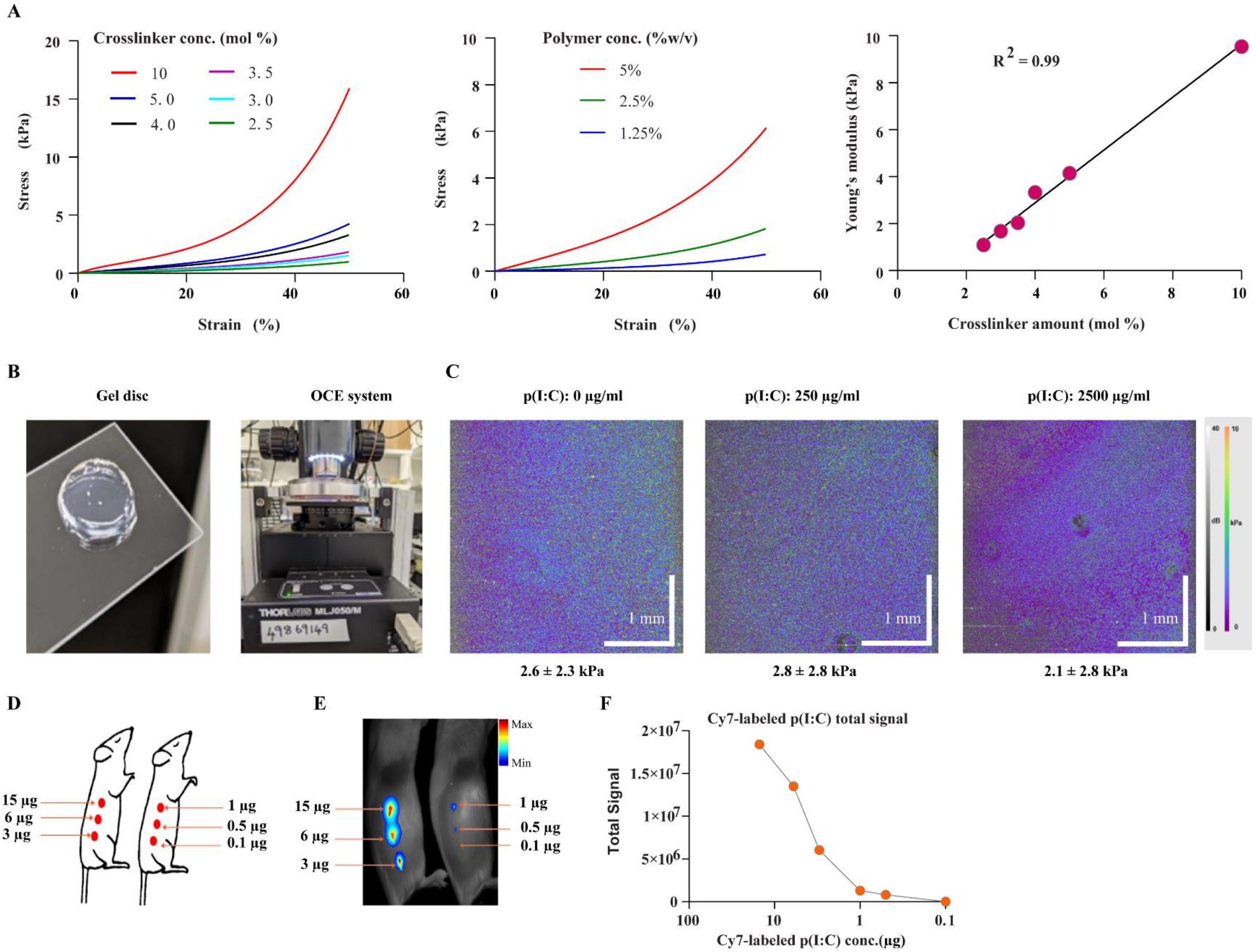

Figure S1. Characterization of HA hydrogels. Related to Figures 1 and 3.

(A) Stress-strain curves of different formulations of hydrogels with varying amount of DTPH crosslinker or hyaluronic acid polymer as well a the correlation of Young's modulus with an increased amount of cross-linker (mol %) while the amount of polymer is kept constant. The experiment was performed once. N = three replicates for each hydrogel.

(B) Sample of a gel disc and the optical coherence elastography system as previously described by Kennedy *et al*. [1].

(C) Representative quantitative micro-elastography (QME) scan of hydrogels with different amount of encapsulated poly(I:C), with stiffness calculated in kPa. Representative QME images of gels with poly(I:C) concentrations of 0 µg/ml, 250 µg/ml, and 2500 µg/ml, are shown in the lateral (xy) plane, acquired over a lateral field of view of 3 mm × 3 mm, approximately 200 µm below the top surface of the gel. The lateral voxel size of 3 µm × 3 µm resulted in 1,000,000 elasticity measurements in each 2-D image. In addition, the mean and standard deviation of all the elasticity measurements over the QME field of view was quantified for each poly(I:C) concentration. The elasticity mean ± standard deviation for the 0 µg/ml, 250 µg/ml, and 2500 µg/ml was 2.6 ± 2.3 kPa, 2.8 ± 2.8 kPa, and 2.1 ± 2.8 kPa, respectively. Data are presented as mean ± SD.

(D-F) *In vivo* quantification of Cy7 labeled poly(I:C). (D) Experimental design. Mice were injected s.c. with different amounts (final volume 50 µl) of Cy7-labeled poly(I:C), at adjacent points.

(E) Fluorescence IVIS imaging showing the Cy7 signal from labeled poly(I:C) injected in mice.

(F) Quantification of Cy7 signal showing the detection limit of Cy7-labeled poly(I:C).

The experiment was performed once. In (F), the Cy7 signal was quantified as total signal (x10^6 phot/cm2/s).

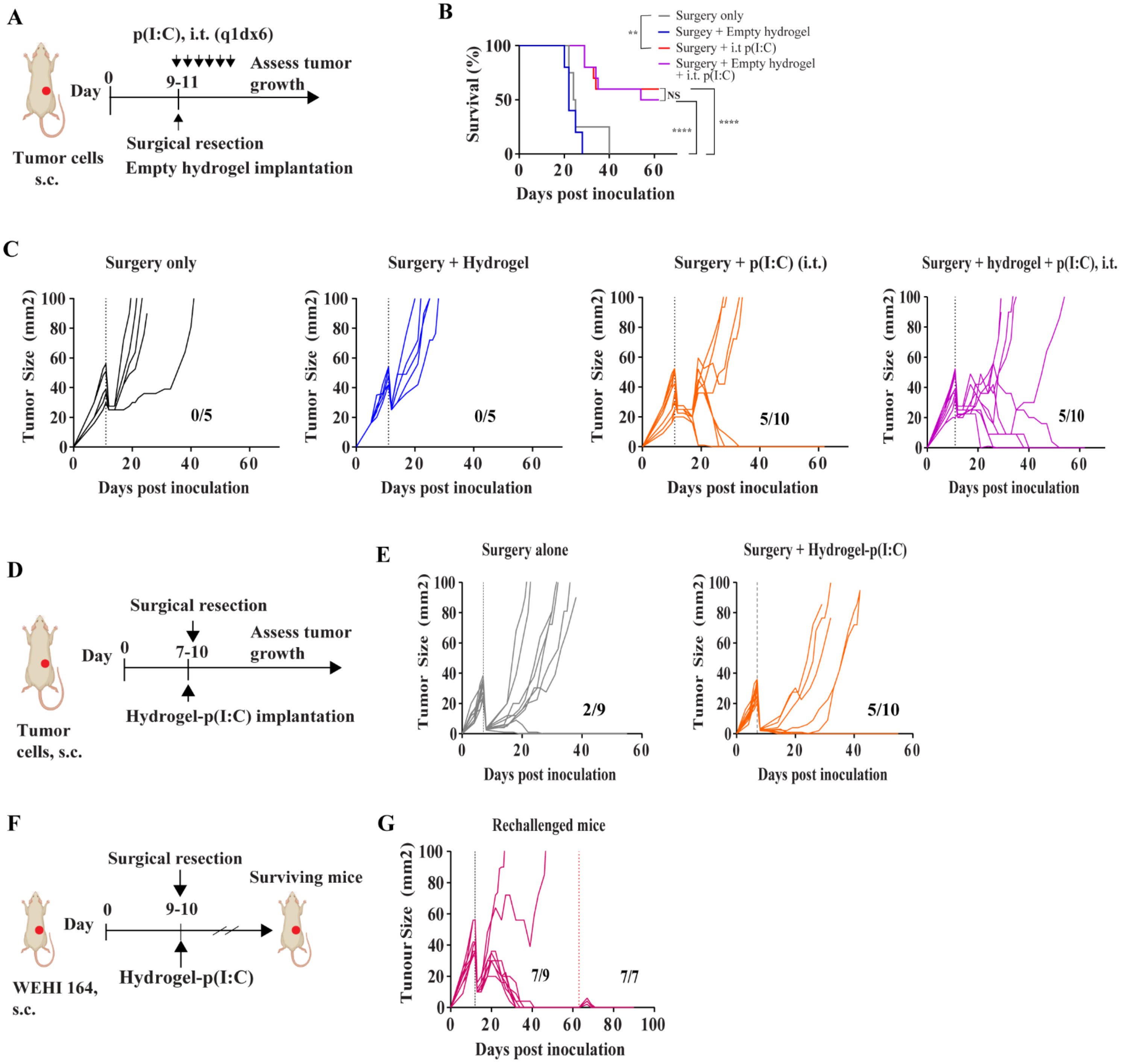

Figure S2. Efficacy of poly(I:C)-releasing hydrogel. Related to Figure 3.
(A and B) Intraoperative hydrogel application does not impair poly(I:C) efficacy**.** (A) Experimental design. Mice were inoculated s.c. with WEHI 164 tumor cells on the right flank. Once tumors were established, a 50% debulk of the tumor was performed and 100 µl of empty hydrogel was left in the resection site. Mice were dosed with poly(I:C) or vehicle, daily for 6 days (q1dx6) in the tumor/wound area.
(B and C) Survival curves (B) and tumor growth curves (C) of WEHI 164-bearing mice showing different treatment groups. The dashed line indicates the day of surgery. NS: non-significant. N = 5-10 mice per group. Statistical analyzes were performed using log-rank (Mantel-Cox) test to compare survival. The significance is represented with asterisks (*) according to the following values. *$P \leq 0.05$, **$P \leq 0.005$, ***$P \leq 0.0005$, ****$P \leq 0.0001$.
(D and E) Efficacy of intraoperative poly(I:C)-releasing hydrogel in M3-9-M tumor model**.** (D) Experimental design. Mice were inoculated with M3-9-M tumor cell line, s.c, on the right flank. Once tumors were established, a 90% debulk of tumor was performed and 100 µl of empty hydrogel or hydrogel-p(I:C) (250 µg) was left in the tumor resection site. (E) Tumor growth curves. The dashed line indicates the day of surgery. Data shown are from 2 independent experiments. N = 9-10 mice per group.
(F and G) The intraoperative poly(I:C)-releasing hydrogel induces a systemic immune memory response.
(F) Experimental design as in Figure 3D. Surviving, tumor-free, mice were rechallenged with WEHI 164 tumor cell line, s.c. on the left flank (four weeks after complete tumor regression).
(G) Tumor growth curves of surviving mice before and after rechallenge. The dashed black line indicates the day of surgery. The dashed red line indicates the day of rechallenge. Data shown is from one experiment, n = 7-9 mice per group.

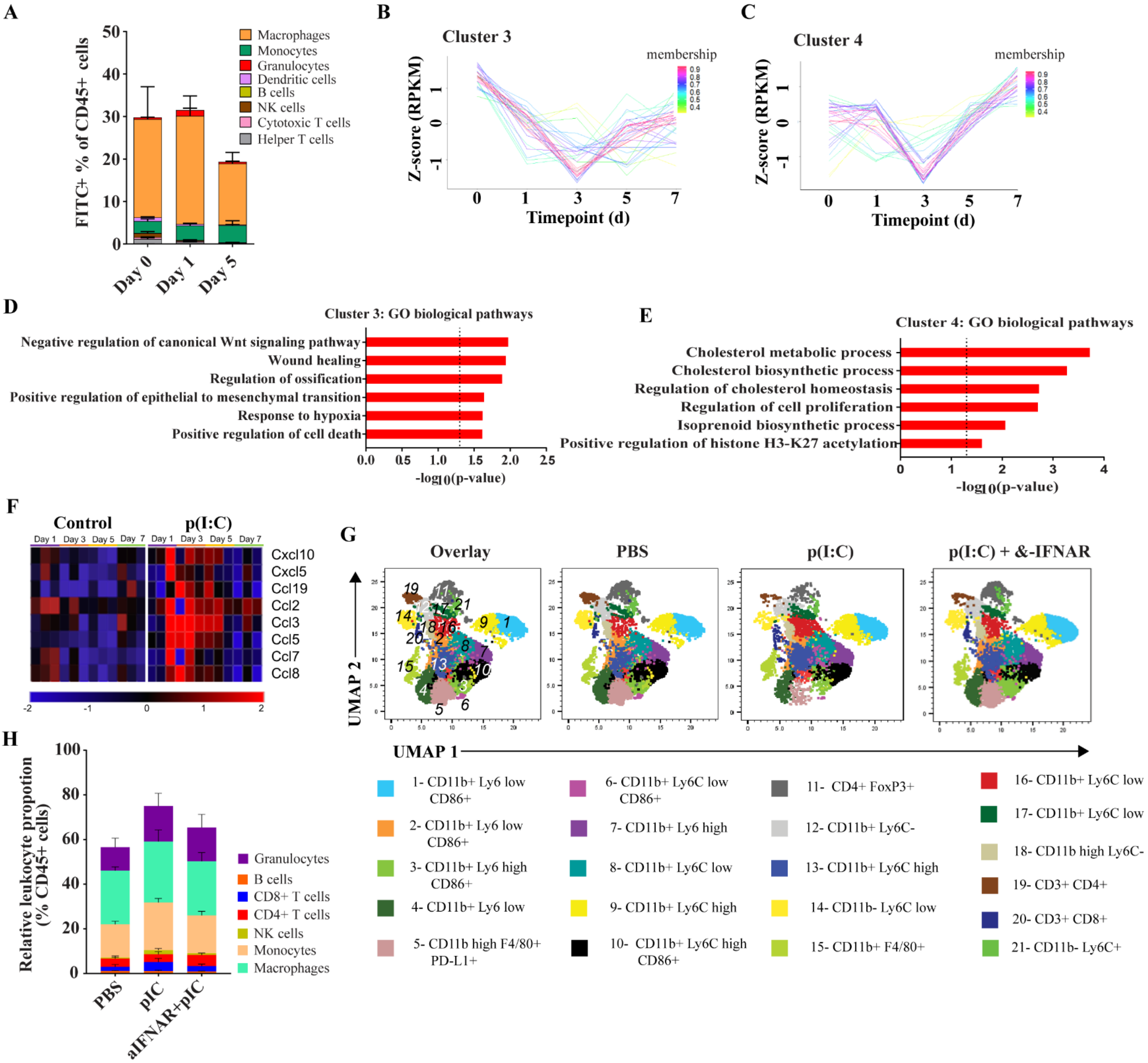

Figure S3. Prolonged poly(I:C) administration induces a transient IFNα response and reshapes the TME. Related to Figure 4.
(A) Uptake of poly(I:C) by immune cells. Experimental setup as in Figure 4A. Mice bearing WEHI 164 tumors were treated with poly(I:C), i.t., daily, for 4 days and a single injection of fluorescein-labeled poly(I:C) (50 ug), i.t., one hour before harvesting tumors for flow cytometry. Bar chart for FITC-labeled poly(I:C) detection in respective populations. N = 3 mice per group.
(B-E) Time-dependent analysis of gene expression in poly(I:C) or vehicle treated tumors. Experimental setup as in Figure 4C. Mice with established WEHI 164 tumors were treated with daily poly(I:C), 10 ug/day, or vehicle, i.t., for 6 days (q1dx6). Tumors were harvested on days 1, 3, 5, and 7, immediately placed in RNA later before subsequent RNA extraction for bulk RNA sequencing. N = 3 mice per group.
(B) Time course variation in expression of gene clusters for Cluster 3. (C) Time course variation in expression of gene clusters for Cluster 4. TCseq analysis was used to cluster genes with similar expression over time.
(D) Top 5 upregulated biological pathways in Cluster 3.
(E) Top 5 upregulated biological pathways in Cluster 4.
(F) Poly(I:C) induces the expression of monocyte-attracting chemokines in the tumor microenvironment. Experimental setup as in Figure 4C. Heatmap of gene expression levels of monocyte-attracting chemokines in poly(I:C) and vehicle treated groups across the different time points. N = 3 mice per group.
(G) UMAP showing clustering of cell populations across different treatment groups.
(H) Relative proportion of different immune cells across different treatement groups.

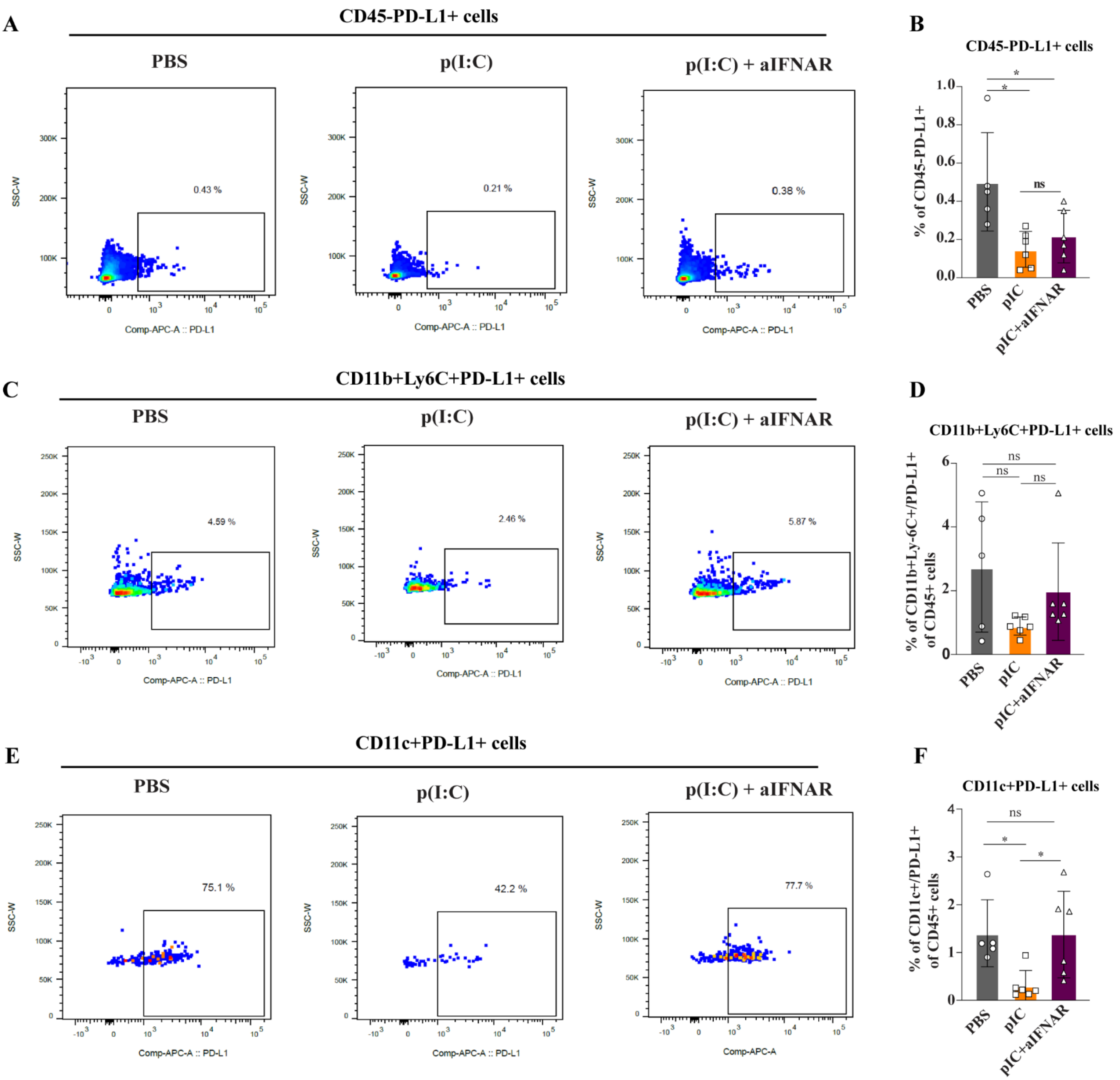

Figure S4. Expression of PD-L1 across different cell populations in the TME. Related to Figure 4 and Figure 5.
Experimental setup as in Figure 4I.
(A) Representative FACS plots of PD-L1 expression, gated on $CD45^-$/Live cells.
(B) Proportion (%) of $PD\text{-}L1^+$ cells within the $CD45^-$ cell fraction.
(C) Representative FACS plots of PD-L1 expression, gated on $CD11b^+Ly6C^+$ cells.
(D) Proportion (%) of $CD11b^+Ly6C^+$ $PD\text{-}L1^+$ cells within $CD45^+$ cell fraction.
(E) Representative FACS plots of PD-L1 expression, gated on $MHCII^+CD11c^+$ cells.
(F) Proportion (%) of $CD11c^+$ $PD\text{-}L1^+$ cells within $CD45^+$ cell fraction.
N = 5-6 biologically independent samples per group. Statistical analyzes were performed with one-way ANOVA followed by Tukey's multiple comparison test, ns = not significant. The significance is represented with asterisks (*) according to the following values: *P≤ 0.05, **P≤ 0.005, ***P≤ 0.0005, ****P≤ 00001.

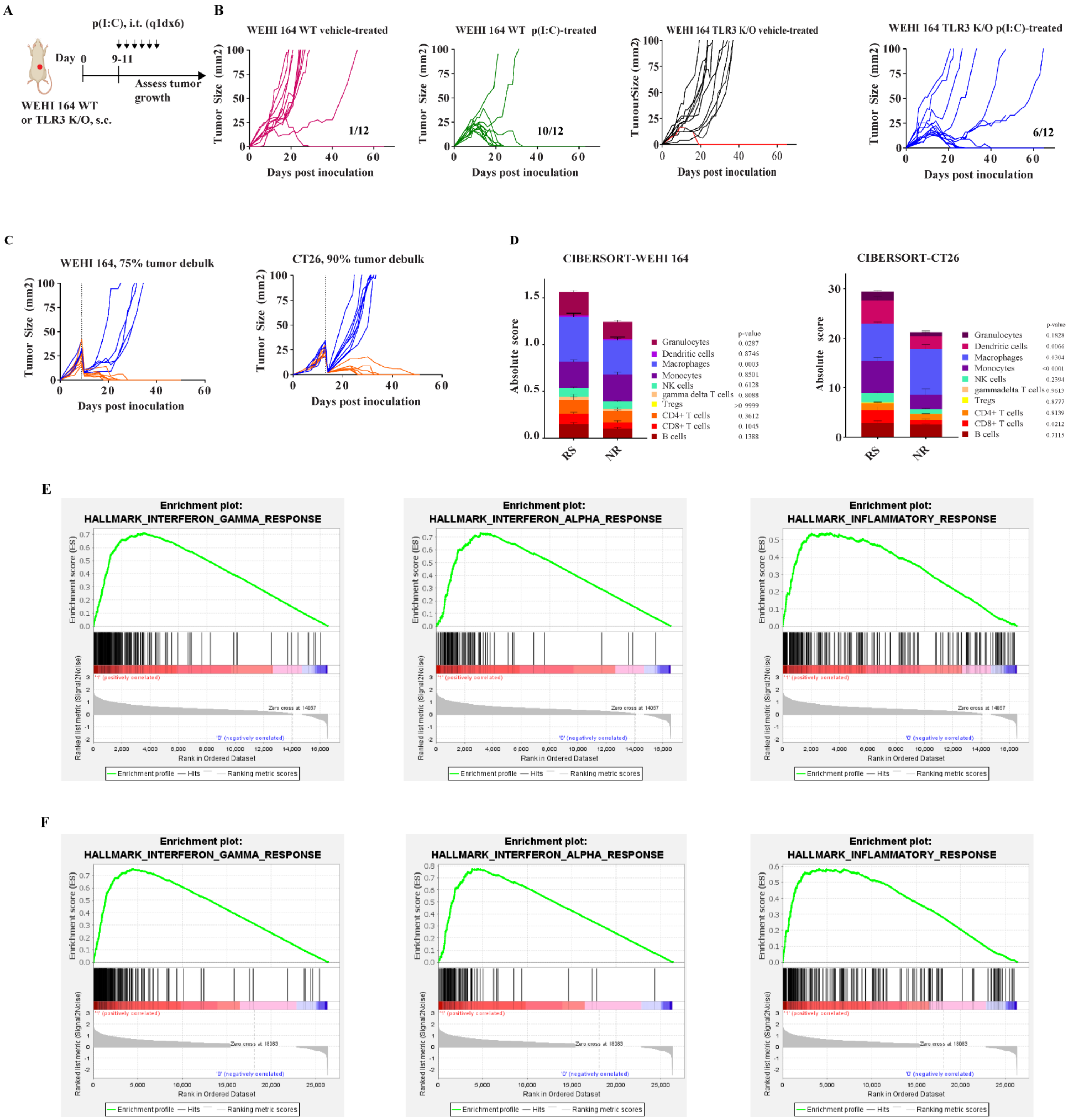

Figure S5. A pre-existing IFN gene signature predicts response to poly(I:C) hydrogel. Related to Figure 5.

(A and B) Efficacy of poly(I:C) in tumors derived from TLR3 K/O WEHI 164 cells. (A) Experimental setup. Mice were inoculated with either wild type WEHI 164 or TLR3 K/O WEHI 164 cells, s.c, on the right flank. Mice with established tumors were treated with poly(I:C), 10 µg/day for 6 days (q1dx6). (B) Tumor growth curves of TLR3 K/O WEHI 164- or WEHI 164 WT-bearing mice. Data shown are pooled from 2 independent experiments, n = 8-10 mice per group. WT: wild type. TLR3 K/O: Toll like receptor 3 knock out.

(C) Tumor growth curves and CIBERSORT plots of responders and non-responders in WEHI 164 and CT26 tumors treated with poly(I:C) hydrogel, allowing classification into responders (orange) or non-responders (blue). N = 9-10 mice per treated group.

(D) CIBERSORT plots from RNAseq data of responsive and non-responsive WEHI 164 and CT26 tumors. N = 4-6 biologically independent samples per group. For CIBERSORT data, statistical analysis was performed using two-way ANOVA with Benjamini and Hochberg correction for multiple comparisons.

(E and F) Responsive tumors are characterized by an IFN-active, inflammatory microenvironment. Experiment setup as in Figure 5C. (E) GSEA plots comparing responsive versus non-responsive WEHI 164 tumors. N = 5 biologically independent samples per group. (F) GSEA plots comparing responsive versus non-responsive CT26 tumors. N = 4-6 biologically independent samples per group. The experiment was performed once. GSEA software was used to analyze normalized gene expression data to compare responders and non-responders.

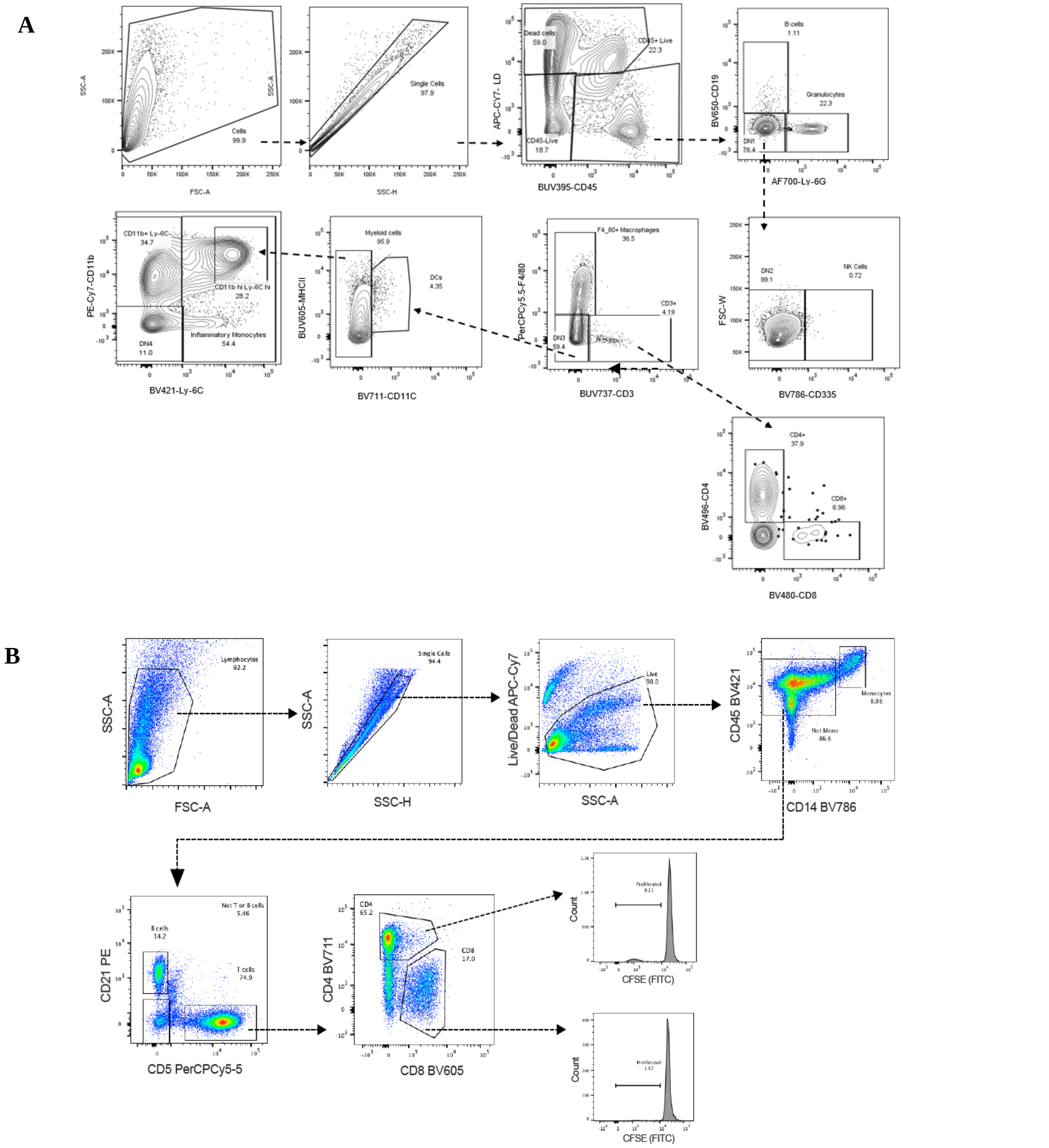

Figure S6. Gating strategies. Related to Figure 4 and Figure 6.
(A) Gating strategy for murine immune cell populations. Live CD45^{+} cells, DN1 (excludes CD19^{+} B cells, Ly6G^{+} Granulocytes), DN2 (excludes CD335^{+} NK cells), F4/80+ macrophages, CD11c^{+} DCs, CD11b^{+} Ly6^{hi} cells, CD3^{+} cells, CD4^{+} and CD8^{+} T cells gating strategies are indicated.
(B) Gating strategy for canine immune cell populations. Live CD45^{+} cells, CD4^{+} and CD8^{+} T cells, and CFSE proliferation gating strategies are indicated.

Supplemental Reference

1. Kennedy, K.M., Chin, L., McLaughlin, R.A., Latham, B., Saunders, C.M., Sampson, D.D., and Kennedy, B.F. (2015). Quantitative microelastography: Imaging of tissue elasticity using compression optical coherence elastography. Sci. Rep. 5, 15538. https://doi.org/10.1038/srep15538.
